# Effect of Antioxidants on the Gut Microbiome Profile and Brain Functions: A Review of Randomized Controlled Trial Studies

**DOI:** 10.3390/foods14020176

**Published:** 2025-01-08

**Authors:** Aleksandra Hyży, Hanna Rozenek, Ewa Gondek, Mariusz Jaworski

**Affiliations:** 1 Department of Education and Research in Health Sciences, Faculty of Health Sciences, Medical University of Warsaw, 00-518 Warsaw, Poland; aleksandra.hyzy@wum.edu.pl; 2 Department of Health Psychology, Medical University of Warsaw, 00-518 Warsaw, Poland; hanna.rozenek@wum.edu.pl (H.R.); mariusz.jaworski@wum.edu.pl (M.J.); 3 Department of Food Engineering and Process Management, Warsaw University of Life Sciences, 02-787 Warsaw, Poland

**Keywords:** antioxidants, microbiome, brain function, berries, papaya, plant food

## Abstract

Background: Antioxidants are widely recognized for their potential health benefits, including their impact on cognitive function and gut microbiome modulation. Understanding these effects is essential for exploring their broader clinical applications. Objectives: This review aims to evaluate the effects of antioxidants on the gut microbiome and cognitive function, with a focus on findings from randomized controlled trials (RCTs). Methods: The studies involved human participants across a range of age groups, with interventions encompassing natural antioxidant sources, such as berries, as well as specific antioxidant vitamins. An extensive search across PubMed, SCOPUS, and Web of Science databases identified six relevant RCTs, each evaluated for potential bias. Results: These studies focused on a variety of antioxidant-rich products, including both naturally derived sources and supplemental forms. Antioxidants, including vitamins C, B2, and D, along with polyphenols such as xanthohumol, fermented papaya, peanuts, and berry extracts, demonstrate the potential to support cognitive function and promote gut health through mechanisms that modulate microbiome diversity and reduce inflammation. However, observed changes in microbiome diversity were modest and inconsistent across the studies. Conclusions: While preliminary evidence suggests that antioxidants may benefit gut health and cognitive function, the heterogeneity of existing studies limits their immediate clinical applicability. Additionally, more robust RCTs are needed to substantiate these findings and guide future interventions.

## 1. Introduction

In recent years, the impact of the gut microbiome on human health, especially brain function, has garnered increasing attention [1]. The microorganisms inhabiting the gut participate in a range of biochemical processes that regulate the gut–brain axis, influencing neurotransmission, inflammation, and the integrity of the blood–brain barrier [2]. It has been noted that diet is a key factor affecting the state of the gut microbiome, with specific dietary components capable of modulating its composition and functions [3].

Preliminary studies on animal models and in vitro experiments suggest that diets rich in fiber, polyphenols, and compounds with anti-inflammatory or antioxidant properties support microbiome diversity and health [4,5,6]. In particular, antioxidants—compounds abundantly found in fruits, vegetables, nuts, and spices—have attracted researchers’ interest due to their potential role not only in reducing oxidative stress but also in modulating the gut microbiome [7]. Antioxidants may influence the gut microbiome through several mechanisms, including altering the redox environment in the gut [8], modulating the activity of gut bacteria involved in fermentation and short-chain fatty acid production [9,10], and reducing gut inflammation [11], which can favor the growth of beneficial microbial species. These changes may indirectly affect brain function by improving neurotransmitter balance and blood–brain barrier integrity [12].

However, numerous challenges remain in fully understanding the impact of antioxidants on the human body. One major issue is the biological and chemical diversity of antioxidants, which adds complexity to their actions and complicates the prediction of therapeutic effects. Antioxidants encompass a wide range of compounds, from vitamins such as vitamin C and E, to polyphenolic compounds present in plants, as well as endogenous compounds [13], including antioxidant enzymes like superoxide dismutase, and non-enzymatic antioxidants such as glutathione. It is important to clarify that superoxide dismutase is not a direct antioxidant but an antioxidant enzyme that plays a crucial role in cellular defense mechanisms. Each of these groups has unique properties, distinct mechanisms of action, and different biochemical pathways, making it challenging to generalize the effects of individual antioxidants [14]. Furthermore, the activity of antioxidant enzymes can be influenced by dietary intake [15], particularly of foods rich in polyphenols, which are known to modulate cellular antioxidant capacity.

Additionally, the effects of antioxidants are highly dose-dependent. At lower doses, antioxidants can exhibit protective effects, supporting the body’s repair and defense mechanisms. However, at higher doses, some antioxidants may display pro-oxidative activity, leading to an excess of free radicals and potentially damaging cells. This type of effect has been documented with certain polyphenolic compounds and supplements containing high doses of vitamin E and beta-carotene [13,16,17]. Moreover, the antioxidant and pro-oxidative activities of specific compounds are not solely dose-dependent but may also vary significantly between different organisms due to interspecies differences in metabolism, enzyme systems, bioavailability, and oxidative status [18,19]. For example, certain plant extracts and isolated polyphenols can function as antioxidants in one species while demonstrating pro-oxidative properties in another. These differences underscore that determining optimal doses and understanding the mechanisms responsible for the shift from protective to harmful effects are key challenges in antioxidant research.

In addition to interspecies variability, the local environment, such as pH [20], reactive oxygen species (ROS) concentration [21], and the presence of other bioactive compounds, can further modulate the activity of antioxidants. Some antioxidants may behave differently under varying oxidative stress conditions, exhibiting protective effects in systems with moderate stress levels but contributing to pro-oxidative damage in environments characterized by excessive oxidative pressure. These dynamic interactions highlight the need for more comprehensive studies that consider not only the dose but also the biological context and environmental factors to better understand the dual roles of antioxidants [22,23].

Moreover, the heterogeneity of antioxidants means that their bioavailability and stability can vary depending on the form of administration (e.g., oral or intravenous) and source (e.g., supplements or natural foods). Antioxidants present in foods often act synergistically with other nutrients, whereas isolated supplements may not demonstrate the same efficacy and can even produce different health effects. Since there is no single, universal mechanism of action for antioxidants, their effectiveness and potential risks are difficult to predict and depend on the specific physiological and metabolic conditions of individuals being studied [24,25].

While studies on antioxidant supplementation’s effects on the gut microbiome and brain functions are growing, findings remain inconsistent due to methodological differences and population variability [26,27]. Most evidence comes from preclinical studies conducted in vitro and on animal models, primarily rats and mice, in which neurodegenerative diseases such as Alzheimer’s or Parkinson’s are induced in a controlled manner. While these models provide valuable insights into the biochemical and physiological mechanisms underlying gut–brain axis interactions, translating these findings to the human population remains a major challenge. This is partly due to interspecies differences in metabolism and the complexity of the human microbiome, which means that results from animal models may not fully reflect therapeutic effects or potential side effects in humans. Bridging preclinical results with clinical practice requires further human research to verify the efficacy and safety.

The aim of this review is to summarize clinical studies (RCTs) on the effects of antioxidants on the gut microbiome and brain functions, considering both potential mechanisms of action and research limitations.

## 2. Materials and Methods

### 2.1. Search Strategy

A literature search was conducted in the PubMed, Web of Science, and SCOPUS databases, covering publications up to 31 October 2024. The following keywords were used: (“Polyphenols” OR “Antioxidants” OR “Flavonoids” OR “Resveratrol” OR “Epicatechin” OR “Curcumin” OR “Quercetin” OR “Catechin” OR “Lycopene” OR “Lutein” OR “Zeaxanthin” OR “Anthocyanins” OR “Ellagic Acid” OR “Proanthocyanidins”) AND (“Gut Microbiota” OR “Intestinal Microbiome” OR “Gut-Brain Axis” OR “Microbiome Composition”) AND (“Brain Health” OR “Cognitive Function” OR “Mental Health” OR “Neuroprotection”) AND (“Human” OR “Clinical Trial” OR “Human Study” OR “Intervention”). The search strategy also included MeSH terms to ensure comprehensive retrieval. Additionally, reference lists of included studies were manually searched to identify additional eligible studies. Details of the search procedure are provided in the Supplementary Materials—Table S1.

### 2.2. Study Selection

Three researchers independently reviewed the titles and abstracts to identify potentially relevant studies. This was carried out to select studies that might be significant for further analysis. Full-text articles were then obtained and assessed for eligibility according to the inclusion criteria by all authors. The following inclusion criteria were applied: studies published up to 31 October 2024, studies involving human participants, randomized controlled trials (RCTs), and studies available in English. While preparing the literature review, we used the Rayyan software (version 2024) to manage the scientific references.

### 2.3. Population

The review included studies on humans across all age groups. The intervention involved the use of various antioxidants. Comparison groups included either a placebo or no intervention at all. Intervention details covered the type of product containing antioxidant substances used, the administered dose, and the duration of treatment.

### 2.4. Primary Outcomes

The review analyzed various primary outcomes, depending on the objective of the RCT. Below are the main indicators measured in the studies:

Cognitive Function and Neurological Health:

(a) Endothelial function assessed through flow-mediated dilation (FMD);
(b) Cognitive function measured by a set of specialized tests;
(c) Stress reactivity;
(d) Brain-derived neurotrophic factor (BDNF) levels;
(e) Other biochemical markers, including tryptophan, kynurenine, and the tryptophan/kynurenine ratio.

Inflammatory and Antioxidant Biomarkers:

(a) Inflammatory markers (e.g., IL-1β and IL-10);
(b) Oxidative stress and antioxidant biomarkers, such as cortisol, superoxide dismutase (SOD), malondialdehyde (MDA), and protein carbonyl content (PCC).

Gut Microbiome Composition and Health:

(a) Gut microbiome composition and diversity;
(b) Microbiome metabolism and metabolites;
(c) Gut health markers (e.g., short-chain fatty acids, inflammatory markers, enterocyte damage, and bacterial translocation).

### 2.5. Study Design

The studies considered in this review comprised randomized controlled trials (RCTs). Any disagreements between reviewers were resolved through discussion or, when necessary, consultation with a senior third reviewer.

### 2.6. Study Selection and Data Extraction

The selection process involved three independent reviewers who screened titles and abstracts to identify relevant studies. Full-text articles were then obtained and evaluated according to predefined inclusion criteria. Data extraction was conducted by one reviewer and subsequently verified by three additional reviewers to ensure accuracy. The extracted data included key study characteristics (author, year, and study type), general participant information (e.g., age, gender, and selected health parameters), and intervention details (type of antioxidant product, dose, and duration), as well as outcome measures related to cognitive function and changes in the microbiome.

### 2.7. Risk-of-Bias Assessment

In this review, we assessed the risk of bias. For randomized controlled trials (RCTs), we applied Cochrane criteria, which evaluate bias across five key areas: the randomization process, deviations from intended interventions, missing outcome data, outcome measurements, and selection of reported results [28]. Discrepancies were resolved through discussion.

## 3. Results

**Figure 1:**
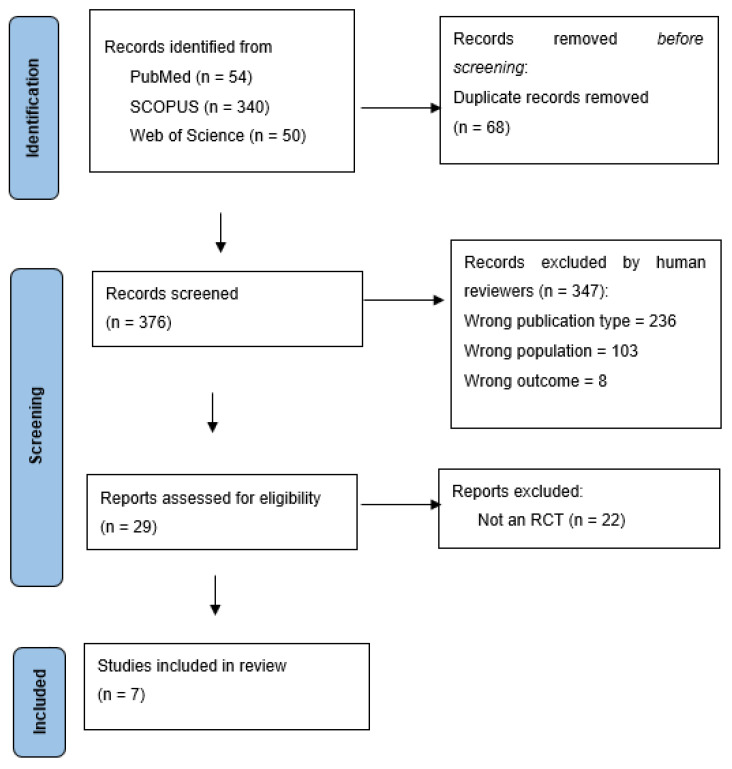
PRISMA flow diagram of studies selected.

The literature search initially identified 444 articles. After a thorough screening process, 29 papers were selected for full-text review. Of these, 21 papers were excluded for not meeting the inclusion criteria, resulting in 7 studies being included in this review, as illustrated in Figure 1.

### 3.1. Risk-of-Bias Assessment

The table presents the risk-of-bias assessment for seven articles across the five main domains of the Cochrane RoB 2 tool: the randomization process, deviations from intended interventions, missing outcome data, outcome measurement, and selection of the reported result. Each domain for each study is rated at levels of risk: “low”, “some concerns”, or “high”, allowing for the determination of the overall risk-of-bias level [28].

The analysis of these seven articles for risk of bias indicates that, although most studies were rated at a low risk level, some ambiguities warrant consideration. The most common areas of potential bias were missing outcome data and selection of the reported outcome. This suggests that incomplete data and selective reporting may introduce some risk of distortion in interpreting the results (Table 1).

### 3.2. Study Characteristics and Groupings

#### 3.2.1. General Characteristics of Included Studies

The analyzed studies focused on evaluating various antioxidant-rich products, including both natural food sources in plants [29,30,31,34,35] and supplements [33] (Table 2). The review included berry fruits such as wild blueberries and black currants, which are rich in polyphenols, particularly anthocyanins, known for their anti-inflammatory and neuroprotective effects [29,30]. Studies also examined fermented papaya, a product with high antioxidant activity achieved through biofermentation processes, which may support gut barrier integrity [31]. Two studies analyzed the impact of peanut consumption on cognitive health [34,35]. Additionally, xanthohumol—a polyphenol derived from hops—was studied for its potential to modulate the gut microbiome and improve cognitive function [32]. The review also considered supplementation with selected antioxidant vitamins, such as vitamins C, B2, and D, which contribute to oxidative balance in the body and support cognitive resilience [33]. Each of these products was analyzed for its unique role in regulating the gut microbiome and its impact on brain health, enabling the identification of potential benefits and limitations associated with their use in cognitive function support and prevention.

The analyzed studies included diverse groups of participants (Table 1). In six studies, the participants were healthy adults [29,30,32,33,34,35] of varying ages, selected to assess the preventive effects associated with antioxidant supplementation in the daily diet. One study focused on healthy older adults to investigate the impact of natural antioxidant sources, such as berries, on supporting cognitive function and gut microbiome health in a group particularly vulnerable to cognitive decline. One study involved patients with neurodegenerative diseases [31], such as Parkinson’s disease, to evaluate the potential benefits of antioxidant supplementation as part of supportive therapy.

#### 3.2.2. Interventions

The interventions analyzed included both natural sources of antioxidants in plants and their supplemental forms, with varying durations to assess short- and long-term effects on the microbiome and cognitive functions (Table 3). For natural sources, such as berries [29,30], interventions lasted from 4 to 8 weeks, aiming to observe their impact on microbiome composition and support cognitive health in both healthy participants and older adults. Another natural source of antioxidants analyzed was peanuts. In this case, the intervention lasted 6 months [34,35]. In the case of vitamins C, B2, and D and xanthohumol, these were administered over a period of 8 to 12 weeks [32,33].

### 3.3. Effects on Cognitive Functions

The analyzed components, including berries [29,30], peanuts [34,35], fermented papaya [31], hop flavonoids [32], and antioxidant vitamins [33], exhibit a mix of similar and diverse effects on cognitive functions. Many of these interventions share common pathways, such as reducing oxidative stress, improving redox status, enhancing cerebral blood flow, strengthening gut–brain communication, supporting neuroprotection, and regulating mood through SCFA production. While these interventions often share common pathways, the mechanisms and outcomes can vary based on the specific compound and context.

Polyphenol-rich foods such as berries [29,30] and peanuts [34,35] are particularly notable for their ability to enhance memory, mood, and cognitive performance. These benefits are attributed to increased cerebral blood flow, improved endothelial function, and the modulation of neuroprotective pathways. For example, the polyphenols found in blueberries and blackcurrants are linked to enhanced neurogenesis [29,30], while peanut-derived resveratrol and hydroxybenzoic acids reduce anxiety and improve memory through their anti-inflammatory and antioxidant properties [34,35].

Fermented papaya operates through distinct systemic pathways, improving oxidative balance and reducing damage to cellular components such as DNA and proteins. While its effects are more systemic than localized to the brain, the reduction in oxidative stress indirectly supports brain health by mitigating neuroinflammatory processes [31]

Hop flavonoids, such as xanthohumol, show potential for cognitive benefits through their interaction with the gut–brain axis. These flavonoids have been associated with improved glucose and fat metabolism, which may influence brain energy regulation. However, the variability in cognitive outcomes across studies highlights the importance of individual microbiome profiles in mediating these effects [32].

Antioxidant vitamins, including vitamins C, B2, and D, enhance cognitive function through various mechanisms. Vitamin C, for example, has been shown to reduce oxidative stress in brain tissues, support neurotransmitter synthesis, and improve mental clarity. Vitamin D influences cognitive outcomes through its interaction with the vitamin D receptor (VDR), which regulates gene expression involved in brain function and neuroprotection. A combined treatment of vitamins C and B2 has demonstrated synergistic effects, further improving mood and memory in clinical trials [33].

While there are shared benefits across these components, including reductions in oxidative stress and improvements in endothelial function, the magnitude and mechanisms of their effects vary significantly. This variability underscores the complexity of cognitive enhancement and the need to consider individual factors such as age, baseline health, and dietary patterns when designing interventions.

### 3.4. Effects on the Gut Microbiome

The analyzed components exhibit diverse effects on the gut microbiome, reflecting their unique biochemical properties and interaction mechanisms (Table 4).

Polyphenol-rich foods such as berries and peanuts generally promote beneficial bacterial populations, such as Bifidobacterium and Akkermansia, and enhance the production of short-chain fatty acids (SCFAs), including butyrate and acetate. These metabolites are known to improve gut barrier integrity and reduce systemic inflammation, indirectly influencing other health outcomes [29,30].

Fermented papaya, on the other hand, exerts minimal direct effects on microbiome diversity but may support gut health through indirect pathways. It enhances antioxidant defenses and reduces oxidative stress, potentially stabilizing gut function and preventing inflammation-associated conditions like “leaky gut syndrome [31].

Hop flavonoids, such as xanthohumol, demonstrate highly variable effects depending on individual baseline microbiome composition. For example, participants with a Prevotella-dominant enterotype show greater shifts in microbial composition compared to those with a Bacteroides-dominant profile. This variability underscores the role of personalized nutrition in microbiome-targeted interventions [32].

Antioxidant vitamins, including vitamins C, B2, and D, influence the gut microbiota through mechanisms like pH modulation, oxidative stress reduction, and microbial cross-feeding. Vitamin C, in particular, significantly increases microbial diversity and enhances SCFA production, promoting a balanced gut ecosystem. The synergistic effects of combined vitamins (e.g., C and B2) further highlight their potential for optimizing microbiome modulation [33].

Collectively, while some components share overlapping benefits—such as SCFA production and modulation of beneficial bacterial populations—their impacts differ in magnitude and scope. This diversity points to the importance of understanding individual and intervention-specific factors to tailor strategies for gut microbiome health.

### 3.5. Heterogeneity of Studies

The analyzed studies exhibited significant heterogeneity in terms of interventions, participant characteristics, and research objectives. The study objectives covered a wide range of topics, including the effects of polyphenol supplementation (e.g., anthocyanins from blueberries [29] or blackcurrants [30]) on cognitive functions, and the impact of vitamins delivered to the colon to modulate the microbiome. The characteristics of the populations were equally diverse—ranging from healthy young adults (18–33 years old) to elderly individuals with neurodegenerative diseases, which involved varying health profiles, ages, and lifestyles. The number of participants ranged from 27 to 96, potentially affecting the statistical power of individual analyses.

The interventions varied considerably in terms of ingredients, doses, and durations, which ranged from 4 weeks to 6 months. The effects of the interventions on cognitive functions were inconsistent, spanning from significant improvements in short-term memory to no differences observed between the placebo and intervention groups. Similar variability was observed for microbiome outcomes; some studies reported increased microbial diversity or changes in specific bacterial taxa, while others found no significant differences. The diversity of analytical methods and outcome measures further complicates the comparability of results.

The heterogeneity in study populations, interventions, and outcomes underscores the need for cautious interpretation of results and limitations in generalizing conclusions. This analysis highlights the necessity for future studies with more uniform designs to develop consistent recommendations.

## 4. Discussion

### 4.1. Summary of Key Findings

**Figure 2:**
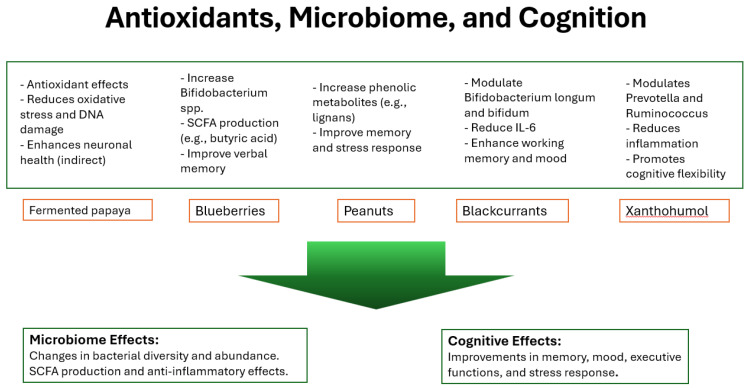
Antioxidants, microbiome, and cognition.

The conducted review of RCT studies on antioxidant supplementation suggests its potentially beneficial impact on cognitive functions and the gut microbiome, although results in these areas are varied and require further analysis [29,30,31,32,33,34,35] (Figure 2).

#### 4.1.1. General Conclusions from Studies in the Context of Cognitive Functions

RCT studies analyzing the impact of antioxidants on cognitive functions indicate their beneficial effects, particularly in improving working memory and overall mental performance. For instance, improvements in cognitive test scores were observed following supplementation with flavonoid-rich blackcurrant drinks [29] or wild blueberry powder [30], particularly in populations prone to cognitive decline, such as older adults. Rather than repeating specific numerical results, we highlight the broader implications of these findings, such as the potential for targeted dietary interventions to address age-related cognitive challenges.

Similarly, studies on peanut consumption revealed notable enhancements in memory, which were attributed to their polyphenol content. Additionally, the inclusion of antioxidant vitamins showed promising effects on cognitive resilience through pathways such as oxidative stress reduction and neuroprotection, offering insights into the role of micronutrient supplementation in cognitive health maintenance [34,35].

While these findings collectively suggest that antioxidant supplementation can enhance cognitive functions, variations in study outcomes underscore the influence of individual differences, such as baseline health status, dietary patterns, and genetic predispositions [33].

#### 4.1.2. General Conclusions from Studies in the Context of the Microbiome

The impact of antioxidants on the gut microbiome varied across studies, with some interventions showing significant changes in microbial diversity and SCFA production, while others yielded more modest results. For example, while polyphenol-rich foods such as berries and peanuts generally promoted beneficial bacterial populations, interventions like fermented papaya showed minimal direct microbiome effects, instead contributing to overall gut health through oxidative stress reduction.

These results suggest that while antioxidants may influence the gut microbiome, their effects are limited and may depend on specific metabolic conditions and the baseline composition of the microbiome.

Vitamin supplementation provided more consistent results, with increased levels of beneficial bacteria like Akkermansia and Faecalibacterium and enhanced SCFA production. However, heterogeneity in the interventions, population characteristics, and methodologies highlights the need for standardized protocols in future research.

Rather than reiterating detailed findings from individual studies, we emphasize the overarching patterns and identify key mechanisms, such as SCFA-mediated gut–brain communication and microbiota modulation, as central to understanding the role of antioxidants in gut health.

Mechanically, antioxidants support the production of SCFAs, such as butyrate, propionate, and acetate, which are key metabolites produced by the gut microbiota. SCFAs exert protective effects on the gut barrier by stimulating mucin production and the expression of tight junction proteins, such as occludin and claudin, thereby enhancing barrier integrity and reducing endotoxin translocation, which lowers systemic inflammation [36,37]. In the context of the gut–brain axis, SCFAs modulate microglial activity in the brain, potentially reducing neuroinflammation and supporting neurogenesis in the hippocampus [9,38]. Antioxidants may also influence the redox balance in the gut, promoting microbiome diversity and fostering the growth of SCFA-producing bacteria, such as Faecalibacterium prausnitzii and Akkermansia muciniphila [39,40].

Animal studies have demonstrated that diets rich in polyphenols can enhance SCFA production and modulate gut microbiota composition. For instance, studies in rodent models have shown that polyphenols from berries increase the abundance of beneficial bacterial species, such as Faecalibacterium and Akkermansia, contributing to improved gut barrier function and reduced neuroinflammation [41].

### 4.2. Critical Evaluation of Study Design and Heterogeneity

The reviewed studies exhibit significant variability in terms of intervention types, participant characteristics, and methodological approaches, which challenges the generalization of results and underscores the complexity of studying antioxidant supplementation. This heterogeneity stems from differences in study objectives, durations, and outcomes assessed.

One notable limitation across studies is the lack of uniformity in participant inclusion criteria. For example, while some studies targeted specific groups, such as older adults or patients with neurodegenerative diseases, others included a broader population of healthy individuals. This variation in baseline characteristics complicates direct comparisons and limits the applicability of findings to the general population. Moreover, the exclusion criteria, such as the absence of chronic conditions or the use of specific medications, may have inadvertently excluded subpopulations that could benefit most from these interventions.

Intervention durations varied considerably, ranging from 4 weeks to 6 months. Short-term studies primarily assessed immediate changes in cognitive functions and microbiome composition, while longer studies offered insights into sustained effects and the potential for adaptation over time. However, the relatively short duration of most studies limits the understanding of long-term impacts, particularly for microbiome stability and the persistence of cognitive benefits. Future studies with extended intervention periods are essential to elucidate these effects.

Another significant source of heterogeneity is the variability in antioxidant dosages and delivery formats (e.g., whole foods, extracts, or supplements). The analysis of the study results indicates that the effectiveness of antioxidant doses depends on both the type of intervention and its objective. In the study by Gillies et al. [30], a daily dose of 300 mL of blueberry juice over four weeks significantly improved working memory by 33%, making this intervention the most effective in enhancing cognitive function. On the other hand, the study by Wood et al. [29] demonstrated that regular consumption of polyphenol-rich berries over 12 weeks led to an increase in beneficial gut bacteria, highlighting the efficacy of this intervention in improving microbiome health. Other studies, such as supplementation with probiotics or fermented papaya, showed less pronounced effects, particularly in the context of microbiome modulation. These findings emphasize the importance of tailoring the dose and type of antioxidants to specific health objectives, such as cognitive enhancement or microbiome improvement.

Additionally, methodological differences in microbiome analysis, such as the choice of sequencing platforms, bioinformatics pipelines, and microbiome metrics, further limit cross-study comparability. While some studies focused on specific taxa or functional groups, others employed broader diversity indices, making it difficult to draw unified conclusions. Harmonizing methodologies and incorporating functional analyses, such as SCFA quantification or metabolomic profiling, would enhance the interpretability and relevance of findings.

To address the challenges posed by the heterogeneity of study populations, interventions, and outcomes, future RCTs should consider the following recommendations:

(a) Standardization of inclusion criteria: Establishing clear and uniform participant selection criteria, such as specific age ranges, baseline dietary habits, and health conditions, will improve comparability across studies.
(b) Harmonization of methodologies: Adopting standardized tools for microbiome analysis (e.g., sequencing platforms and bioinformatics pipelines) and cognitive assessment protocols will enhance the reliability of results.
(c) Longer intervention and follow-up periods: Extending the duration of studies will allow researchers to assess the persistence and long-term effects of interventions on the gut microbiome and cognitive health.
(d) Larger, more diverse study populations: Increasing sample sizes and including diverse demographic groups will ensure that findings are generalizable to broader populations.
(e) Stratified analyses: Conducting subgroup analyses based on baseline characteristics, such as microbiome composition or oxidative stress levels, will help identify population-specific effects and optimize intervention strategies.

Lastly, small sample sizes in many studies reduce statistical power and increase the risk of type II errors, potentially obscuring subtle but clinically meaningful effects. To mitigate these issues, future research should prioritize larger cohorts and stratified analyses based on baseline characteristics, such as microbiome composition or oxidative stress levels. Such approaches would not only improve the robustness of results but also enable the development of personalized intervention strategies.

### 4.3. The Role of Individual Differences

While the reviewed studies highlight the potential benefits of antioxidant supplementation, it is essential to consider individual differences that may influence these outcomes. Genetic factors play a crucial role, as polymorphisms in genes involved in oxidative stress response, such as those encoding antioxidant enzymes (e.g., superoxide dismutase or glutathione peroxidase) [42,43], can modulate the effectiveness of antioxidant interventions. For instance, individuals with specific genetic variants may exhibit enhanced or diminished responses to certain antioxidants, which could explain the variability observed in the study results.

Additionally, dietary habits significantly affect antioxidant absorption and metabolism. Baseline dietary intake of antioxidants from natural food sources may influence the incremental benefits of supplementation [44,45]. For example, individuals with diets already rich in fruits and vegetables may derive less benefit from additional antioxidant supplements compared to those with low baseline intake. Furthermore, differences in dietary patterns may interact with gut microbiome composition, affecting the bioavailability and efficacy of supplemented antioxidants.

In animal models, individual differences in microbiome composition have been shown to significantly influence responses to polyphenol supplementation [46,47]. For example, animals with microbiomes dominated by specific bacterial species exhibited more pronounced cognitive improvements following antioxidant interventions, highlighting the importance of baseline microbial profiles in modulating gut–brain interactions.

Lifestyle factors, including physical activity, smoking, and alcohol consumption, also impact the oxidative stress burden and the body’s response to antioxidants [46]. For instance, smokers or individuals with sedentary lifestyles may experience greater oxidative stress, potentially increasing their responsiveness to antioxidant supplementation. Conversely, high levels of physical activity may enhance endogenous antioxidant capacity, modifying the need for external supplementation.

Considering these individual differences is critical for designing personalized antioxidant interventions. Future studies should aim to stratify participants based on genetic markers, dietary intake, and lifestyle factors to better understand the nuanced effects of antioxidants on health outcomes.

### 4.4. Research Gaps and Directions for Future RCT Studies

The analysis of current studies highlights significant gaps and potential directions for future research on the impact of supplementation with berries [29], blackcurrants [30], hops [32], and peanuts [34,35] on health, cognitive functions, and the gut microbiome. There is a need for more in-depth and complex studies to fully understand the mechanisms of action of these substances and their effectiveness in a health context.

In the case of berries, studies suggest that polyphenols found in blueberries may have beneficial effects on vascular and cognitive functions. However, it remains unclear how butyrate—a metabolite produced by the gut microbiome—affects these processes. Research in rodent models also highlights the potential for specific polyphenols to modulate neurogenesis in the hippocampus via gut–brain axis pathways [48], such as SCFA production and neuroinflammation reduction. Therefore, future studies should focus on analyzing the role of butyrate in mediating the effects of blueberry polyphenols. Moreover, researchers note a high variability in individual responses to blueberry interventions, potentially due to differences in the gut microbiome and other biological factors. Larger studies are therefore necessary to confirm previous findings and gain a better understanding of the mechanisms underlying these effects [29].

Blackcurrant, as a rich source of flavonoids, also attracts researchers’ attention. It is suggested that targeted modification of the gut microbiome—such as by profiling the microbiome at the study’s start or through concurrent probiotic use—could enhance the benefits of flavonoid supplementation. Additionally, larger interventional studies including a variety of polyphenol sources are needed to better examine the role of the microbiome in processing these substances and maximizing their health effects. It is also important that future studies analyze polyphenol metabolites present in participants’ urine and stool and correlate them with clinical outcomes to enhance understanding of these compounds’ mechanisms [30].

Current research on the effects of peanut consumption on cognitive health and stress response highlights significant research gaps. One of the key areas for further exploration is the role of phenolic metabolites derived from gut microbiota in the observed improvements in memory and stress reduction.

Animal studies have provided valuable insights into the molecular mechanisms through which phenolic metabolites derived from peanut polyphenols influence brain health [49]. For instance, these studies suggest that these metabolites enhance neurovascular health and modulate inflammatory pathways, laying the groundwork for more targeted clinical research.

A detailed understanding of the molecular mechanisms through which these metabolites influence cognitive health, including their interactions with enzymes, transcription factors, and cerebral blood flow, remains missing. A notable limitation of existing studies is that most of the literature is based on animal models, emphasizing the need for more human studies to draw more meaningful conclusions regarding the gut–brain axis. Additionally, constraints such as the small sample sizes underscore the necessity of future research involving larger cohorts, longer durations, and more rigorous methodological approaches. Future studies should focus on elucidating the molecular mechanisms of phenolic metabolites, expanding the scope of clinical research on peanut products, and incorporating extended follow-up periods in interventional studies.

Supplementation with hops [32], particularly xanthohumol (XN), opens additional possibilities in the context of gut microbiome modulation. Studies indicate that higher doses of XN may have a significant impact on the microbiome; however, further research is required to fully understand these interactions. Future research should involve larger human cohorts to gain better insights into the interactions between xanthohumol and the gut microbiome and to explore the potential of this compound as a targeted microbiome therapy.

## 5. Conclusions

In summary, antioxidant supplementation, including vitamins C, B2, and D, as well as polyphenols such as xanthohumol, fermented papaya, peanuts, and berry extracts, shows the potential to enhance cognitive function and gut health by modulating gut microbiome diversity and reducing inflammation. These antioxidants may support the gut–brain axis primarily by increasing the production of short-chain fatty acids (SCFAs) and improving gut barrier integrity, which helps mitigate oxidative stress—a factor often associated with cognitive decline and neurodegenerative diseases.

Based on the review of studies conducted, it appears that such supplements may be particularly beneficial for individuals experiencing heightened oxidative stress or inflammation, which impacts both cognitive function and gut health. However, the available evidence remains preliminary and shows considerable variability in study methodologies, population characteristics, types of supplements, and durations of interventions, limiting their direct clinical applicability.

To enhance the practical applicability of these findings, we propose the following clinical practice recommendations:

- Targeted Supplementation: Antioxidant supplementation should be prioritized for individuals with known oxidative stress, systemic inflammation, or early signs of cognitive impairment. Such populations may benefit most from interventions targeting the gut–brain axis.
- Dietary Integration: Encouraging the inclusion of natural antioxidant-rich foods—such as berries, nuts, and fermented products—can provide a sustainable approach to support gut and cognitive health.
- Monitoring and Personalization: Clinicians should consider monitoring biomarkers of oxidative stress and inflammation to guide personalized supplementation strategies. Tailoring interventions to individual microbiome profiles may improve the outcomes.
- Multidisciplinary Approach: Collaboration between healthcare providers, dietitians, and researchers is essential to integrate these findings into patient care, ensuring evidence-based and individualized recommendations.

Further, more targeted research is necessary to determine optimal dosages and understand the synergistic effects between various antioxidants and microbiome profiles, which could ultimately lead to the development of personalized supplementation strategies that support cognitive health through microbiome modulation.

Ultimately, the realization of such research requires strengthening interdisciplinary collaboration among the fields of nutrition, microbiology, and neuroscience. Integrating their findings can significantly advance research on the gut–brain axis, thereby contributing to improvements in public health.

## Figures and Tables

**Table 1:** Risk-of-bias assessment.

Study	Bias Domain					Overall Bias
	Bias Arising from the Randomization Process	Bias due to Deviations from the Intended Interventions	Bias due to Missing Outcome Data	Bias in Measurement of the Outcome	Bias in Selection of the Reported Result	
Wood et al. [29]	low	some concerns	low	some concerns	low	some concerns
Gillies et al. [30]	low	low	low	some concerns	low	low
Bolner et al. [31]	low	some concerns	low	low	low	low
Jamieson et al. [32]	low	low	some concerns	low	low	low
Pham et al. [33]	low	low	low	low	low	low
Parilli-Moser et al. [34]	low	some concerns	low	low	low	low
Parilli-Moser et al. [35]	low	some concerns	low	low	low	low

**Table 2:** General characteristics of analyzed RCT studies.

Authors	Year	Study Objectives	Microbiome Analysis	Cognitive Function Analysis	Participant Characteristics	Sample Size
Wood et al. [29]	2023	To assess the daily intake of WBB (poly)phenols and its effects on vascular health and cognitive abilities in healthy older adults.	Yes	Yes	- Participants were healthy adults aged 65–80 years. - BMI ranged between 18 and 35 kg/m^2^. - Excluded individuals with heart disease, diabetes, metabolic disorders, cognitive issues, allergies, or certain medications. - Female participants were postmenopausal and not on HRT.	66 randomized at baseline (35 in wild blueberry group, 31 placebo); 61 completed.
Gillies et al. [30]	2024	To explore the influence of a 4-week FBB supplement compared to a placebo on brain and mental health markers, biochemical data, and gut microbiota structure and function. To evaluate how initial microbiota composition correlates with outcomes.	Yes	Yes	- Healthy women aged 18–45 years. - BMI of 18–30 kg/m^2^. - No substance abuse or significant alcohol consumption. - Excluded those with psychiatric conditions or major medical issues. - Majority held university degrees and engaged in regular physical activity.	40 enrolled; 38 completed both intervention phases.
Bolner et al. [31]	2023	(1) To evaluate the effects of FPP on oxidative stress in Parkinson’s disease (PD). (2) To investigate its impact on clinical and gut microbiota parameters in PD patients.	Yes	Yes	- Participants were adults with Parkinson’s. - 82% male. - Age range: females 52–72 years; males 57–79 years. - Blood biochemical and hematological data were assessed.	Total: 39 (19 FPP, 20 placebo).
Jamieson et al. [32]	2024	(1) To assess the impact of an 8-week XN intervention on gut microbiota diversity. (2) To explore how the gut microbiome influences XN metabolism in healthy adults.	Yes	No	- Healthy adults aged 21–50 years. - Gender-balanced groups. - Participants distributed as 14 in the placebo group and 16 in the XN group.	30 enrolled; 27 completed (13 placebo, 14 XN).
Pham et al. [33]	2021	To investigate the impact of colon-targeted delivery of vitamins A, B2, C, D, and E on gut microbiota using human trials and in vitro experiments. To study effects on intestinal barrier integrity and immune function.	Yes	No	- Healthy adults with no chronic or acute conditions. - Excluded individuals on specific medications or dietary regimens. - Limited demographic details reported.	96 participants.
Parilli-Moser et al. [34]	2021	To assess how regular peanut consumption (skin-roasted peanuts and peanut butter) influences cognitive performance and short-chain fatty acid (SCFA) levels.	Yes	Yes	- Healthy adults aged 18–33 years (average 22.7). - Mostly female participants (70%). - Free from chronic illnesses.	63 participants (19 men, 44 women).
Parilli-Moser et al. [35]	2023	To extend the 2021 study by investigating the production of microbial phenolic metabolites (MPMs) and the role of gut microbiota in metabolizing peanut-derived polyphenols.	Yes	Yes	- Healthy adults aged 18–33 years (average 22.7). - Mostly female participants (70%). - Free from chronic illnesses.	63 participants (19 men, 44 women).

Abbreviations: WBB—freeze-dried wild blueberry; BMI—body mass index; HRT—hormone replacement therapy; FBB—flavonoid-rich blackcurrant beverage; FPP—fermented papaya preparation; XN—xanthohumol.

**Table 3:** RCT study characteristics in terms of intervention.

Authors	Intervention Characteristics	Effects on Cognitive Functions	Tests Used to Measure Cognitive Functions	Effects on Microbiome	Duration
Wood et al. [29]	Daily intake of 26 g freeze-dried wild blueberry powder (302 mg anthocyanins).	Improved verbal learning and task-switching accuracy (*p* < 0.05).	Rey’s Auditory Verbal Learning Task (AVLT), Corsi block task, serial 3s and 7s subtraction tasks, task-switching task.	Increased beneficial bacteria (e.g., Ruminiclostridium, Christensenellaceae) with positive correlations to cognitive/vascular benefits.	12 weeks
Gillies et al. [30]	300 mL flavonoid-rich blackcurrant beverage (151 mg anthocyanins, 308 mg total polyphenols, 200 mg L-theanine).	33% improvement in working memory performance (*p* < 0.001); reduction in anger/hostility (37%, *p* = 0.013) and tension/anxiety (20%, *p* = 0.023).	Purple Multitasking Framework (MTF): arithmetic tasks, Stroop test, letter recall, visual tracking.	No significant changes in gut microbiome composition or diversity observed.	4 weeks
Bolner et al. [31]	Fermented papaya preparation (FPP) dietary supplement in a crossover design.	Improvement in cognitive function (MOCA) and quality of life, particularly in Parkinson’s disease patients.	Montreal Cognitive Assessment (MOCA): evaluates memory, attention, executive functions, language, visuospatial abilities, and orientation.	No significant differences in beneficial microbiome changes between FPP and placebo groups.	6 months
Jamieson et al. [32]	24 mg daily xanthohumol (XN) supplementation.	Not analyzed.	Not applicable.	No significant microbiome diversity changes or SCFA production. Certain metabolites observed in specific enterotypes (Prevotella, Ruminococcus).	8 weeks
Pham et al. [33]	Daily vitamin supplementation: combinations of A, B2, C, D3, and E.	Not analyzed.	Not applicable.	Combination of B2 + C decreased Proteobacteria and increased Coprococcus while decreasing Sutterella. Firmicutes increase trend observed.	4 weeks
Parilli-Moser et al. [34]	Three-arm RCT: skin-roasted peanuts (25 g/day—SRP), peanut butter (32 g/day—PB), control butter (32 g/day—CB).	Improved memory (immediate, verbal, total) in SRP and PB groups correlated with polyphenol intake.	Wechsler Memory Scale (WMS-IV), Wechsler Adult Intelligence Scale (WAIS-III, IV), Trail Making Test (TMT), verbal fluency tests.	Increased SCFA levels (e.g., acetic, butyric acids) in SRP and PB groups. No microbiome changes beyond SCFA observed.	6 months
Parilli-Moser et al. [35]	Follow-up to 2021 study: Same intervention groups (SRP, PB, CB).	Significant verbal and overall memory improvement in PB group associated with increased urinary microbial phenolic metabolites (MPMs).	Wechsler Memory Scale (WMS-IV), WAIS-III, IV, TMT, verbal fluency tests, correlation analysis with microbial metabolites.	Higher urinary MPM levels (e.g., hydroxybenzoic acids, enterolignans) in SRP and PB groups compared to CB.	6 months

Abbreviations: SCFA: short-chain fatty acid; RCT: randomized controlled trial; MPM: microbial phenolic metabolite.

**Table 4:** Bacterial strains analyzed in the context of brain functions.

Bacterial Strain	Inflammatory Markers	Tryptophan Production	SCFA Production	Phenolic Metabolites	Product	Study
Bifidobacterium longum	+	+	+	Not analyzed	Blackcurrant	Gillies et al. [30]
Bifidobacterium bifidum	+	Not analyzed	+	Not analyzed	Blackcurrant	Gillies et al. [30]
*Bifidobacterium* spp.	+	+	+	Not analyzed	Wild blueberry	Wood et al. [29]
Prevotella, Ruminococcus	+	Not analyzed	+	Not analyzed	Xanthohumol (XN)	Jamieson et al. [32]
No specific strains	+	Not analyzed	Not analyzed	Not analyzed	Fermented papaya (FPP)	Bolner et al. [31]
No specific strains	+	Not analyzed	Not analyzed	+	Peanuts	Parilli-Moser et al. [34]
No specific strains	+	Not analyzed	Not analyzed	+	Peanuts	Parilli-Moser et al. [35]

Explanations: “+”: effect demonstrated.

## Data Availability

No new data were created or analyzed in this study. Data sharing is not applicable to this article.

## Supplementary Materials

The following supporting information can be downloaded at https://www.mdpi.com/article/10.3390/foods14020176/s1: Table S1: Keywords searched in databases.
