# Supplementary material for: Effect of Antioxidants on the Gut Microbiome Profile and Brain Functions: A Review of Randomized Controlled Trial Studies
Source: Foods. 2025 Jan 8;14(2):176. doi: 10.3390/foods14020176 (PMC11764720; doi:10.3390/foods14020176)
Supplement: Supplementary file 1 [file foods-14-00176-s001.zip › foods-3385337-supplementary.pdf]

Table S1. Keywords searched in databases

PubMed	("Polyphenols" OR "Antioxidants" OR "Flavonoids" OR "Resveratrol" OR "Epicatechin" OR "Curcumin" OR "Quercetin" OR "Catechin" OR "Lycopene" OR "Lutein" OR "Zeaxanthin" OR "Anthocyanins" OR "Ellagic Acid" OR "Proanthocyanidins")<br>AND<br>("Gut Microbiota" OR "Intestinal Microbiome" OR "Gut-Brain Axis" OR "Microbiome Composition") AND ("Brain Health" OR "Cognitive Function" OR "Mental Health" OR "Neuroprotection")<br>AND<br>("Human" OR "Clinical Trial" OR "Human Study" OR "Intervention")
SCOPUS	(TITLE-ABS-KEY("polyphenols" OR "antioxidants" OR "flavonoids" OR "resveratrol" OR "epicatechin" OR "curcumin" OR "Quercetin" OR "Catechin" OR "Lycopene" OR "Lutein" OR "Zeaxanthin" OR "Anthocyanins" OR "Ellagic Acid" OR "Proanthocyanidins"))<br>AND<br>(TITLE-ABS-KEY("gut microbiota" OR "intestinal microbiome" OR "gut-brain axis" OR "microbiome composition"))<br>AND<br>(TITLE-ABS-KEY("brain health" OR "cognitive function" OR "mental health" OR "neuroprotection"))<br>AND<br>(LIMIT-TO(DOCTYPE, "ar") OR LIMIT-TO(DOCTYPE, "re"))
Web of Science	TS=("polyphenols" OR "antioxidants" OR "flavonoids" OR "resveratrol" OR "epicatechin" OR "curcumin" OR "Quercetin" OR "Catechin" OR "Lycopene" OR "Lutein" OR "Zeaxanthin" OR "Anthocyanins" OR "Ellagic Acid" OR "Proanthocyanidins")<br>AND<br>TS=("gut microbiota" OR "intestinal microbiome" OR "gut-brain axis" OR "microbiome composition")<br>AND<br>TS=("brain health" OR "cognitive function" OR "mental health" OR "neuroprotection")<br>AND<br>TS=("clinical trial" OR "human study" OR "intervention")

Article	Year of publication	Notes	
Hunt, T. and Pontifex, M.G. and Vauzour, D.; (Poly)phenols and brain health – beyond their antioxidant capacity; FEBS Letters	2024	Excluded	Wrong publication type

Liu J and Wang Y and Wang Z and Hao Y and Bai W and Wang Z and Wang J; 5-Heptadecylresorcinol, a Biomarker for Whole Grain Rye Consumption, Ameliorates Cognitive Impairments and Neuroinflammation in APP/PS1 Transgenic Mice.; Molecular nutrition & food research	2020	Excluded	Wrong population
Kumari, A. and Bharadvaja, N.; A comprehensive review on algal nutraceuticals as prospective therapeutic agent for different diseases; 3 Biotech	2023	Excluded	Wrong publication type
Zhao D and Simon JE and Wu Q; A critical review on grape polyphenols for neuroprotection: Strategies to enhance bioefficacy.; Critical reviews in food science and nutrition	2020	Excluded	Wrong publication type
Sip, S. and Rosiak, N. and Sip, A. and Żarowski, M. and Hojan, K. and Cielecka-Piontek, J.; A Fisetin Delivery System for Neuroprotection: A Co-Amorphous Dispersion Prepared in Supercritical Carbon Dioxide; Antioxidants	2024	Excluded	Wrong publication type
Li, Q. and Liang, J. and Fu, N. and Han, Y. and Qin, J.; A Ketogenic Diet and the Treatment of Autism Spectrum Disorder; Frontiers in Pediatrics	2021	Excluded	Wrong outcome
Yegin, Z. and Sudagidan, M.; A medical and molecular approach to kefir as a therapeutic agent of human microbiota: A review; International Journal for Vitamin and Nutrition Research	2024	Excluded	Wrong publication type
Tripathi, A.K. and Mishra, S.K.; A review article on neuroprotective, immunomodulatory, and anti-inflammatory role of vitamin-D3 in elderly COVID-19 patients; Egyptian Journal of Neurology, Psychiatry and Neurosurgery	2023	Excluded	Wrong publication type
Wen, X. and Wang, Z. and Liu, Q. and Lessing, D.J. and Chu, W.; Acetobacter pasteurianus BP2201 alleviates alcohol-induced hepatic and neuro-toxicity and modulate gut microbiota in mice; Microbial Biotechnology	2023	Excluded	Wrong population

Zhang, M. and Niu, H. and Li, Q. and Jiao, L. and Li, H. and Wu, W.; Active Compounds of Panax ginseng in the Improvement of Alzheimer's Disease and Application of Spatial Metabolomics; Pharmaceuticals	2024	Excluded	Wrong publication type
Fuertes, A. and Pérez-Burillo, S. and Apaolaza, I. and Vallès, Y. and Pilar Francino, M. and Rufián-Henares, J.Á. and Planes, F.J.; Adaptation of the human gut microbiota metabolic network during the first year after birth; Frontiers in Microbiology	2019	Excluded	Wrong publication type
Venable KE and Lee CC and Francis J; Addressing Mental Health in Rural Settings: A Narrative Review of Blueberry Supplementation as a Natural Intervention.; Nutrients	2024	Excluded	Wrong publication type
Han, N. and Wen, Y. and Liu, Z. and Zhai, J. and Li, S. and Yin, J.; Advances in the roles and mechanisms of lignans against Alzheimer's disease; Frontiers in Pharmacology	2022	Excluded	Wrong publication type
Cao, Q.-Q. and Lin, L.-X. and Xu, T.-T. and Lu, Y. and Zhang, C.-D. and Yue, K. and Huang, S.-C. and Dong, H.-J. and Jian, F.-C.; Aflatoxin B1 alters meat quality associated with oxidative stress, inflammation, and gut-microbiota in sheep; Ecotoxicology and Environmental Safety	2021	Excluded	Wrong population
Lee, D.W. and Ryu, Y.-K. and Chang, D.-H. and Park, H.-Y. and Go, J. and Maeng, S.-Y. and Hwang, D.Y. and Kim, B.-C. and Lee, C.-H. and Kim, K.-S.; Agathobaculum butyriciproducens Shows Neuroprotective Effects in a 6-OHDA-Induced Mouse Model of Parkinson's Disease; Journal of Microbiology and Biotechnology	2022	Excluded	Wrong population
Alam, J. and Kalash, A. and Hassan, M.I. and Rahman, S.Z.; Agents at the Peak of US FDA Approval for the Treatment of Alzheimer's Disease; Neurological Research	2024	Excluded	Wrong publication type
Kovtun, A.S. and Averina, O.V. and Poluektova, E.U. and Kostyuk, G.P. and	2020	Included	Not a RCT

Danilenko, V.N.; Altered neurometabolic potential of gut microbiome in healthy children of different age; Bulletin of Russian State Medical University			
Panda, S.R. and Chaudhari, V.B. and Ahmed, S. and Kwatra, M. and Jala, A. and Ponneganti, S. and Pawar, S.D. and Borkar, R.M. and Sharma, P. and Naidu, V.G.M.; Ambient particulate matter (PM2.5) exposure contributes to neurodegeneration through the microbiome-gut-brain axis: Therapeutic role of melatonin; Environmental Toxicology and Pharmacology	2023	Excluded	Wrong population
van Zonneveld SM and van den Oever EJ and Haarman BCM and Grandjean EL and Nuninga JO and van de Rest O and Sommer IEC; An Anti-Inflammatory Diet and Its Potential Benefit for Individuals with Mental Disorders and Neurodegenerative Diseases-A Narrative Review.; Nutrients	2024	Excluded	Wrong publication type
Adak, A. and Khan, M.R.; An insight into gut microbiota and its functionalities; Cellular and Molecular Life Sciences	2019	Excluded	Wrong publication type
Álvarez SA and Rocha-Guzmán NE and González-Laredo RF and Gallegos-Infante JA and Moreno-Jiménez MR and Bravo-Muñoz M; Ancestral Food Sources Rich in Polyphenols, Their Metabolism, and the Potential Influence of Gut Microbiota in the Management of Depression and Anxiety.; Journal of agricultural and food chemistry	2022	Excluded	Wrong publication type
Micek, A. and Owczarek, M. and Jurek, J. and Guerrera, I. and Torrisi, S.A. and Grosso, G. and Alshatwi, A.A. and Godos, J.; Anthocyanin-rich fruits and mental health outcomes in an Italian cohort; Journal of Berry Research	2022	Excluded	Wrong population
Xin, M. and Xu, A. and Tian, J. and Wang, L. and He, Y. and Jiang, H. and Yang, B. and Li, B. and Sun, Y.; Anthocyanins as natural bioactives with	2024	Excluded	Wrong publication type

anti-hypertensive and atherosclerotic potential: Health benefits and recent advances; Phytomedicine			
Liaqat, H. and Parveen, A. and Kim, S.-Y.; Antidepressive Effect of Natural Products and Their Derivatives Targeting BDNF-TrkB in Gut–Brain Axis; International Journal of Molecular Sciences	2022	Excluded	Wrong publication type
Zhang, W. and Dong, X. and Huang, R.; Antiparkinsonian effects of polyphenols: A narrative review with a focus on the modulation of the gut-brain axis; Pharmacological Research	2023	Excluded	Wrong publication type
Duan, D. and Chen, M. and Cui, W. and Liu, W. and Chen, X.; Application of probiotics, prebiotics and synbiotics in patients with breast cancer: a systematic review and meta-analysis protocol for randomised controlled trials; BMJ Open	2022	Excluded	Wrong publication type
Du, M.-R. and Zhang, Q.-Y. and Yang, S.-Y. and Chen, J.-Q. and Dan, C.-M. and Lian, L.-D. and Wang, J.; Armillariella tabescens-derived polysaccharides alleviated D-Gal-induced neuroinflammation and cognitive injury through enterocerebral axis and activation of keap-1/Nrf2 pathway; International Journal of Biological Macromolecules	2024	Excluded	Wrong population
do Rosario V and Lorzadeh E and Brodaty H and Anstey KJ and Chan K and Roodenrys S and Kent K and Bliokas V and Phillipson L and Weston-Green K and Francois ME and Jiang X and George J and Potter J and Batterham MJ and Charlton K; Assessing the effect of anthocyanins through diet and supplementation on cognitive function in older adults at risk for dementia: protocol for a randomised controlled trial.; BMJ open	2024	Included	Protocol for RCT
Wei, X. and Xin, J. and Chen, W. and Wang, J. and Lv, Y. and Wei, Y. and Li, Z. and Ding, Q. and Shen, Y. and Xu, X. and Zhang, X. and Zhang, W. and Zu, X.; Astragalus polysaccharide	2023	Excluded	Wrong population

ameliorated complex factor-induced chronic fatigue syndrome by modulating the gut microbiota and metabolites in mice; Biomedicine and Pharmacotherapy			
Parvathy, J. and Sreedevi, A. and Sankaranarayanan, P. and Suchithra, T.V.; Augmented Dark Chocolate with Probiotics: A Lifestyle Approach to Address Symptoms of Parkinson's Disease; Current Pharmacology Reports	2023	Excluded	Wrong publication type
Lyu M and Wang YF and Fan GW and Wang XY and Xu SY and Zhu Y; Balancing Herbal Medicine and Functional Food for Prevention and Treatment of Cardiometabolic Diseases through Modulating Gut Microbiota.; Frontiers in microbiology	2017	Excluded	Wrong publication type
Upadhyay, P. and Tyagi, A. and Agrawal, S. and Kumar, A. and Gupta, S.; Bidirectional Effect of Triphala on Modulating Gut-Brain Axis to Improve Cognition in the Murine Model of Alzheimer's Disease; Molecular Nutrition and Food Research	2024	Excluded	Wrong population
Sun, Z.-Z. and Li, X.-Y. and Wang, S. and Shen, L. and Ji, H.-F.; Bidirectional interactions between curcumin and gut microbiota in transgenic mice with Alzheimer's disease; Applied Microbiology and Biotechnology	2020	Excluded	Wrong population
Syeda, T. and Sánchez-Tapia, M. and Orta, I. and Granados-Portillo, O. and Pérez-Jimenez, L. and Rodríguez-Callejas, J.-D. and Toribio, S. and Silva-Lucero, M.-C. and Rivera, A.-L. and Tovar, A.R. and Torres, N. and Perez-Cruz, C.; Bioactive foods decrease liver and brain alterations induced by a high-fat-sucrose diet through restoration of gut microbiota and antioxidant enzymes; Nutrients	2022	Excluded	Wrong population
Di Meo, F. and Valentino, A. and Petillo, O. and Peluso, G. and Filosa, S. and Crispi, S.; Bioactive polyphenols and neuromodulation: Molecular mechanisms in neurodegeneration;	2020	Excluded	Wrong publication type

International Journal of Molecular Sciences			
Ashique, S. and Mukherjee, T. and Mohanty, S. and Garg, A. and Mishra, N. and Kaushik, M. and Bhowmick, M. and Chattaraj, B. and Mohanto, S. and Srivastava, S. and Taghizadeh-Hesary, F.; Blueberries in focus: Exploring the phytochemical potentials and therapeutic applications; Journal of Agriculture and Food Research	2024	Excluded	Wrong publication type
Mohajeri, M.H.; Brain aging and gut-brain axis; Nutrients	2019	Excluded	Wrong publication type
Weng, M.-H. and Chen, S.-Y. and Li, Z.-Y. and Yen, G.-C.; Camellia oil alleviates the progression of Alzheimer's disease in aluminum chloride-treated rats; Free Radical Biology and Medicine	2020	Excluded	Wrong population
Ditchfield, C. and Kushida, M.M. and Mazalli, M.R. and Sobral, P.J.A.; Can Chocolate Be Classified as an Ultra-Processed Food? A Short Review on Processing and Health Aspects to Help Answer This Question; Foods	2023	Included	Not a RCT
Leclerc, M. and Dudonné, S. and Calon, F.; Can natural products exert neuroprotection without crossing the blood–brain barrier?; International Journal of Molecular Sciences	2021	Excluded	Wrong publication type
Ma, B.-Q. and Jia, J.-X. and Wang, H. and Li, S.-J. and Yang, Z.-J. and Wang, X.-X. and Yan, X.-S.; Cannabidiol improves the cognitive function of SAMP8 AD model mice involving the microbiota-gut-brain axis; Journal of Toxicology and Environmental Health - Part A: Current Issues	2024	Excluded	Wrong population
Ren, M. and Li, H. and Fu, Z. and Li, Q.; Centenarian-Sourced Lactobacillus casei Combined with Dietary Fiber Complex Ameliorates Brain and Gut Function in Aged Mice; Nutrients	2022	Excluded	Wrong population
Pontifex MG and Malik MMAH and Connell E and Müller M and Vauzour D; Citrus Polyphenols in Brain Health and Disease: Current Perspectives.; Frontiers in neuroscience	2021	Excluded	Wrong publication type

Mao, Q. and Zhang, H. and Zhang, Z. and Lu, Y. and Pan, J. and Guo, D. and Huang, L. and Tian, H. and Ma, K.; Co-decoction of Lilii bulbus and Radix Rehmannia Recens and its key bioactive ingredient verbascoside inhibit neuroinflammation and intestinal permeability associated with chronic stress-induced depression via the gut microbiota-brain axis; Phytomedicine	2024	Included	Not a RCT
Kim, T.Y. and Kim, J.M. and Lee, H.L. and Go, M.J. and Joo, S.G. and Kim, J.H. and Lee, H.S. and Lee, D.Y. and Kim, H.-J. and Heo, H.J.; Codium fragile Suppresses PM2.5-Induced Cognitive Dysfunction by Regulating Gut–Brain Axis via TLR-4/MyD88 Pathway; International Journal of Molecular Sciences	2023	Included	Animals
Caracciolo, B. and Xu, W. and Collins, S. and Fratiglioni, L.; Cognitive decline, dietary factors and gut-brain interactions; Mechanisms of Ageing and Development	2014	Excluded	Wrong publication type
Wu, S. and Zhu, Z. and Chen, M. and Huang, A. and Xie, Y. and Hu, H. and Zhang, J. and Wu, Q. and Wang, J. and Ding, Y.; Comparison of Neuroprotection and Regulating Properties on Gut Microbiota between Selenopeptide Val-Pro-Arg-Lys-Leu-SeMet and Its Native Peptide Val-Pro-Arg-Lys-Leu-Met In Vitro and In Vivo; Journal of Agricultural and Food Chemistry	2023	Excluded	Wrong population
Huang, F. and Wang, Z. and Zhang, Z. and Liu, X. and Liang, Y. and Qian, J. and Tu, J. and Tang, X. and Zhang, C. and Fang, B.; Comprehensive evaluation of the mechanism of Banxia Baizhu Tianma Decoction in ameliorating posterior circulation ischemia vertigo based on integrating fecal short-chain fatty acids and 16S rRNA sequencing; Journal of Pharmaceutical and Biomedical Analysis	2024	Excluded	Wrong publication type

Wang, S. and Xu, C. and Liu, H. and Wei, W. and Zhou, X. and Qian, H. and Zhou, L. and Zhang, H. and Wu, L. and Zhu, C. and Yang, Y. and He, L. and Li, K.; Connecting the Gut Microbiota and Neurodegenerative Diseases: the Role of Bile Acids; Molecular Neurobiology	2023	Excluded	Wrong publication type
Parilli-Moser, I. and López-Solís, R. and Domínguez-López, I. and Vallverdú-Queralt, A. and Hurtado-Barroso, S. and Lamuela-Raventós, R.M.; Consumption of peanut products enhances the production of microbial phenolic metabolites related with memory and stress response: Results from the ARISTOTLE trial; Journal of Functional Foods	2023	Included	RCT
Parilli-Moser, I. and Domínguez-López, I. and Trius-Soler, M. and Castellví, M. and Bosch, B. and Castro-Barquero, S. and Estruch, R. and Hurtado-Barroso, S. and Lamuela-Raventós, R.M.; Consumption of peanut products improves memory and stress response in healthy adults from the ARISTOTLE study: A 6-month randomized controlled trial; Clinical Nutrition	2021	Included	RCT
Pathak, S. and Suhanya, P. and Sushmitha, S. and Murugesan, R. and He, F. and Marotta, F. and Banerjee, A.; Coping with stress related effects on the brain – role of neuro-nutraceuticals and gut microbes; International Journal of Probiotics and Prebiotics	2017	Excluded	Wrong publication type
Shikh, E.V. and Nikolaeva, N.B. and Molchanova, N.B. and Elizarova, E.V.; Correction of gut dysbiosis as a promising direction in the prevention of neuroinflammation and cognitive impairment; Voprosy Pitaniia	2023	Included	Not a RCT
Nie, L. and Xiang, Q. and Lin, Y. and Xu, Y. and Wen, W. and Deng, Y. and Chen, J. and Zhu, X. and Xie, L. and Wu, Z.; Correlation between symptoms and cognitive function changes in patients with primary insomnia and pathways in	2024	Included	Not a RCT

gut microbiota; Biochemistry and Biophysics Reports			
Kavyani M and Saleh-Ghadimi S and Dehghan P and Abbasalizad Farhangi M and Khoshbaten M; Co-supplementation of camelina oil and a prebiotic is more effective for in improving cardiometabolic risk factors and mental health in patients with NAFLD: a randomized clinical trial.; Food & function	2021	Included	Not a RCT
Cai, B. and Zhong, L. and Wang, Q. and Xu, W. and Li, X. and Chen, T.; Curcumin alleviates 1-methyl- 4-phenyl- 1,2,3,6-tetrahydropyridine-induced Parkinson’s disease in mice via modulating gut microbiota and short-chain fatty acids; Frontiers in Pharmacology	2023	Excluded	Wrong population
Zhang, F. and Zhou, Y. and Chen, H. and Jiang, H. and Zhou, F. and Lv, B. and Xu, M.; Curcumin Alleviates DSS-Induced Anxiety-Like Behaviors via the Microbial-Brain-Gut Axis; Oxidative Medicine and Cellular Longevity	2022	Excluded	Wrong population
Lamichhane, G. and Liu, J. and Lee, S.-J. and Lee, D.-Y. and Zhang, G. and Kim, Y.; Curcumin Mitigates the High-Fat High-Sugar Diet-Induced Impairment of Spatial Memory, Hepatic Metabolism, and the Alteration of the Gut Microbiome in Alzheimer’s Disease-Induced (3xTg-AD) Mice; Nutrients	2024	Excluded	Wrong population
Zhu, H. and Zhang, H. and Hou, B. and Xu, B. and Ji, L. and Wu, Y.; Curcumin Regulates Gut Microbiota and Exerts a Neuroprotective Effect in the MPTP Model of Parkinson's Disease; Evidence-based Complementary and Alternative Medicine	2022	Excluded	Wrong publication type
Di Meo, F. and Margarucci, S. and Galderisi, U. and Crispi, S. and Peluso, G.; Curcumin, gut microbiota, and neuroprotection; Nutrients	2019	Excluded	Wrong publication type
Cui C and Han Y and Li H and Yu H and Zhang B and Li G; Curcumin-driven	2022	Excluded	Wrong population

reprogramming of the gut microbiota and metabolome ameliorates motor deficits and neuroinflammation in a mouse model of Parkinson's disease.; Frontiers in cellular and infection microbiology			
Elbatreek, M.H. and Mahdi, I. and Ouchari, W. and Mahmoud, M.F. and Sobeh, M.; Current advances on the therapeutic potential of pinocembrin: An updated review; Biomedicine and Pharmacotherapy	2023	Excluded	Wrong publication type
Dogra N and Jakhmola-Mani R and Potshangbam AM and Buch S and Pande Katare D; CXCR4 as possible druggable target linking inflammatory bowel disease and Parkinson's disease.; Metabolic brain disease	2023	Included	Not a RCT
He, J. and Jin, Y. and He, C. and Li, Z. and Yu, W. and Zhou, J. and Luo, R. and Chen, Q. and Wu, Y. and Wang, S. and Song, Z. and Cheng, S.; Danggui Shaoyao San: comprehensive modulation of the microbiota-gut-brain axis for attenuating Alzheimer's disease-related pathology; Frontiers in Pharmacology	2023	Excluded	Wrong population
Liang, J. and Liu, B. and Dong, X. and Wang, Y. and Cai, W. and Zhang, N. and Zhang, H.; Decoding the role of gut microbiota in Alzheimer's pathogenesis and envisioning future therapeutic avenues; Frontiers in Neuroscience	2023	Excluded	Wrong publication type
Sip, S. and Stasiłowicz-Krzemień, A. and Sip, A. and Szulc, P. and Neumann, M. and Kryszak, A. and Cielecka-Piontek, J.; Development of Delivery Systems with Prebiotic and Neuroprotective Potential of Industrial-Grade Cannabis sativa L.; Molecules	2024	Excluded	Wrong publication type
Balakrishnan, R. and Jannat, K. and Choi, D.-K.; Development of dietary small molecules as multi-targeting treatment strategies for Alzheimer's disease; Redox Biology	2024	Excluded	Wrong publication type

Godos J and Currenti W and Angelino D and Mena P and Castellano S and Caraci F and Galvano F and Del Rio D and Ferri R and Grosso G; Diet and Mental Health: Review of the Recent Updates on Molecular Mechanisms.; Antioxidants (Basel, Switzerland)	2020	Excluded	Wrong publication type
Aucoin M and LaChance L and Cooley K and Kidd S; Diet and Psychosis: A Scoping Review.; Neuropsychobiology	2020	Excluded	Wrong publication type
Ettinger, S.; Diet, Gut Microbiome, and Cognitive Decline; Current Nutrition Reports	2022	Excluded	Wrong publication type
Láng L and McArthur S and Lazar AS and Pourtau L and Gaudout D and Pontifex MG and Müller M and Vauzour D; Dietary (Poly)phenols and the Gut-Brain Axis in Ageing.; Nutrients	2024	Excluded	Wrong publication type
Lefèvre-Arbogast S and Thomas A and Samieri C; Dietary factors and brain health.; Current opinion in lipidology	2022	Excluded	Wrong publication type
Gentile, D and Fornai, M and Pellegrini, C and Colucci, R and Blandizzi, C and Antonioli, L; Dietary flavonoids as a potential intervention to improve redox balance in obesity and related co-morbidities: a review; NUTRITION RESEARCH REVIEWS	2018	Excluded	Wrong publication type
Zhang Y and Cheng L and Liu Y and Zhan S and Wu Z and Luo S and Zhang X; Dietary flavonoids: a novel strategy for the amelioration of cognitive impairment through intestinal microbiota.; Journal of the science of food and agriculture	2023	Excluded	Wrong publication type
Liu, S. and Dai, H. and Wang, R. and Zhang, X.; Dietary flavonoids: Role in preventing neurodegenerative diseases caused by brain aging by modulating the gut microbiota; Food Bioscience	2024	Excluded	Wrong publication type
Ye J and Fan H and Shi R and Song G and Wu X and Wang D and Xia B and Zhao Z and Zhao B and Liu X and Wang Y and Dai X; Dietary lipoic acid alleviates autism-like behavior induced by acrylamide in adolescent mice: the	2024	Excluded	Wrong population

potential involvement of the gut-brain axis.; Food & function			
Mougin, C. and Chataigner, M. and Lucas, C. and Leyrolle, Q. and Pallet, V. and Layé, S. and Bouvret, E. and Dinel, A.-L. and Joffre, C.; Dietary Marine Hydrolysate Improves Memory Performance and Social Behavior through Gut Microbiota Remodeling during Aging; Foods	2023	Included	No plants
Cano, R and Bermúdez, V and Galban, N and Garrido, B and Santeliz, R and Gotera, MP and Duran, P and Boscan, A and Carbonell-Zabaleta, AK and Duran-Agüero, S and Rojas-Gómez, D and González-Casanova, J and Díaz-Vásquez, W and Chacín, M and Dávila, LA; Dietary Polyphenols and Gut Microbiota Cross-Talk: Molecular and Therapeutic Perspectives for Cardiometabolic Disease: A Narrative Review; INTERNATIONAL JOURNAL OF MOLECULAR SCIENCES	2024	Excluded	Wrong publication type
Naomi, R and Yazid, MD and Teoh, SH and Balan, SS and Shariff, H and Kumar, J and Bahari, H and Embong, H; Dietary Polyphenols as a Protection against Cognitive Decline: Evidence from Animal Experiments; Mechanisms and Limitations; ANTIOXIDANTS	2023	Excluded	Wrong population
Chatterjee, A. and Kumar, S. and Roy Sarkar, S. and Halder, R. and Kumari, R. and Banerjee, S. and Sarkar, B.; Dietary polyphenols represent a phytotherapeutic alternative for gut dysbiosis associated neurodegeneration: A systematic review; Journal of Nutritional Biochemistry	2024	Excluded	Wrong publication type
La Rosa G and Lonardo MS and Cacciapuoti N and Muscariello E and Guida B and Faraonio R and Santillo M and Damiano S; Dietary Polyphenols, Microbiome, and Multiple Sclerosis: From Molecular Anti-Inflammatory and Neuroprotective Mechanisms to	2023	Excluded	Wrong publication type

Clinical Evidence.; International journal of molecular sciences			
Serra, D. and Almeida, L.M. and Dinis, T.C.P.; Dietary polyphenols: A novel strategy to modulate microbiota-gut-brain axis; Trends in Food Science and Technology	2018	Excluded	Wrong publication type
Frausto, D.M. and Forsyth, C.B. and Keshavarzian, A. and Voigt, R.M.; Dietary Regulation of Gut-Brain Axis in Alzheimer's Disease: Importance of Microbiota Metabolites; Frontiers in Neuroscience	2021	Excluded	Wrong publication type
Schmidt, N.S. and Lorentz, A.; Dietary restrictions modulate the gut microbiota: Implications for health and disease; Nutrition Research	2021	Excluded	Wrong publication type
Naureen, Z. and Dhuli, K. and Medori, M.C. and Caruso, P. and Manganotti, P. and Chiurazzi, P. and Bertelli, M.; Dietary supplements in neurological diseases and brain aging; Journal of preventive medicine and hygiene	2022	Excluded	Wrong publication type
Kincaid, H.J. and Nagpal, R. and Yadav, H.; Diet-microbiota-brain axis in alzheimer's disease; Annals of Nutrition and Metabolism	2021	Excluded	Wrong publication type
Wang, Y. and Dykes, G.A.; Direct Modulation of the Gut Microbiota as a Therapeutic Approach for Alzheimer's Disease; CNS and Neurological Disorders - Drug Targets	2022	Excluded	Wrong publication type
Zhao, Z. and Cui, D. and Wu, G. and Ren, H. and Zhu, X. and Xie, W. and Zhang, Y. and Yang, L. and Peng, W. and Lai, C. and Huang, Y. and Li, H.; Disrupted gut microbiota aggravates working memory dysfunction induced by high-altitude exposure in mice; Frontiers in Microbiology	2022	Excluded	Wrong population
Escribano, B.M. and Luque, E. and Aguilar-Luque, M. and Feijóo, M. and Caballero-Villarraso, J. and Torres, L.A. and Ramirez, V. and García-Maceira, F.I. and Agüera, E. and Santamaria, A. and Túnez, I.; Dose-dependent S-allyl cysteine ameliorates multiple sclerosis	2017	Excluded	Wrong population

disease-related pathology by reducing oxidative stress and biomarkers of dysbiosis in experimental autoimmune encephalomyelitis; European Journal of Pharmacology			
Mohammadi Zadeh, M. and Dehghan, P. and Eslami, Z.; Effect of date seed (Phoenix dactylifera) supplementation as functional food on cardiometabolic risk factors, metabolic endotoxaemia and mental health in patients with type 2 diabetes mellitus: a blinded randomised controlled trial protocol; BMJ Open	2023	Included	Protocol for RCT
Xu, L. and Zeng, X. and Liu, Y. and Wu, Z. and Zheng, X. and Zhang, X.; Effect of Dendrobium officinale polysaccharides on central nervous system disease: Based on gut microbiota; International Journal of Biological Macromolecules	2023	Excluded	Wrong publication type
Vajdi, M. and Khorvash, F. and Rouhani, M.H. and Ghavami, A. and Clark, C.C.T. and Askari, G.; Effect of inulin supplementation on clinical symptoms, inflammatory and oxidative stress markers in women with migraine: study protocol for a randomized clinical trial; Trials	2023	Included	Protocol for RCT
Bianchi, V.E. and Herrera, P.F. and Laura, R.; Effect of nutrition on neurodegenerative diseases. A systematic review; Nutritional Neuroscience	2021	Excluded	Wrong publication type
Gillies NA and Wilson BC and Miller JR and Roy NC and Scholey A and Braakhuis AJ; Effects of a Flavonoid-Rich Blackcurrant Beverage on Markers of the Gut-Brain Axis in Healthy Females: Secondary Findings From a 4-Week Randomized Crossover Control Trial.; Current developments in nutrition	2024	Included	RCT
Dilmore AH and Martino C and Neth BJ and West KA and Zemlin J and Rahman G and Panitchpakdi M and Meehan MJ and Weldon KC and Blach C and Schimmel L and Kaddurah-Daouk R	2023	Excluded	Wrong outcome

and Dorrestein PC and Knight R and Craft S; Effects of a ketogenic and low-fat diet on the human metabolome, microbiome, and foodome in adults at risk for Alzheimer's disease.; Alzheimer's & dementia : the journal of the Alzheimer's Association			
Gao L and Li J and Zhou Y and Huang X and Qin X and Du G; Effects of Baicalein on Cortical Proinflammatory Cytokines and the Intestinal Microbiome in Senescence Accelerated Mouse Prone 8.; ACS chemical neuroscience	2018	Excluded	Wrong population
Pham, V.T.; Fehlbaum, S.; Seifert, N.; Richard, N.; Bruins, M.J.; Sybesma, W.; Rehman, A.; Steinert, R.E. ; Effects of Colon-Targeted Vitamins on the Composition and Metabolic Activity of the Human Gut Microbiome—A Pilot Study; Gut Microbes	2021	Included	RCT
Mehta, K.K. and Bhat, R. and Markande, A.R.; Effects of gut bacteria and their amyloids on mental health and neurodegeneration in Parkinson's disease; Journal of Applied Biology and Biotechnology	2023	Excluded	Wrong publication type
Shimizu, C. and Wakita, Y. and Inoue, T. and Hiramitsu, M. and Okada, M. and Mitani, Y. and Segawa, S. and Tsuchiya, Y. and Nabeshima, T.; Effects of lifelong intake of lemon polyphenols on aging and intestinal microbiome in the senescence-accelerated mouse prone 1 (SAMP1); Scientific Reports	2019	Excluded	Wrong population
Tahmasbi, F. and Mirghafourvand, M. and Shamekh, A. and Mahmoodpoor, A. and Sanaie, S.; Effects of probiotic supplementation on cognitive function in elderly: A systematic review and Meta-analysis; Aging and Mental Health	2022	Excluded	Wrong publication type
Lv M and Yang S and Cai L and Qin LQ and Li BY and Wan Z; Effects of Quercetin Intervention on Cognition Function in APP/PS1 Mice was Affected	2018	Excluded	Wrong population

by Vitamin D Status.; Molecular nutrition & food research			
Neta, F.I. and de Souza, F.E.S. and Batista, A.L. and Pinheiro, F.I. and Cobucci, R.N. and Guzen, F.P.; Effects of Supplementation with Probiotics in Experimental Models of Alzheimer's Disease: A Systematic Review of Animal Experiments; Current Alzheimer Research	2022	Excluded	Wrong population
Bolner, A. and Bertoldi, L. and Benvenuto, G. and Sattin, E. and Bosello, O. and Nordera, G.; Effects of dietary supplementation with fermented papaya on oxidative stress, symptoms, and microbiome in Parkinson's disease; Functional Foods in Health and Disease	2023	Included	RCT
Bukhari, S.I. and Alfawaz, H. and Al-Dbass, A. and Bhat, R.S. and Moubayed, N.M.S. and Bukhari, W. and Hassan, S.A. and Merghani, N. and Elsamaligy, S. and El-Ansary, A.; Efficacy of Novavit in ameliorating the neurotoxicity of propionic acid; Translational Neuroscience	2020	Excluded	Wrong outcome
El-Ansary, A. and Al-Ayadhi, L.; Effects of Walnut and Pumpkin on Selective Neurophenotypes of Autism Spectrum Disorders: A Case Study; Nutrients	2023	Included	Not a RCT
Xu, Y and Xie, MM and Xue, JS and Xiang, L and Li, YL and Xiao, J and Xiao, GR and Wang, HL; EGCG ameliorates neuronal and behavioral defects by remodeling gut microbiota and TotM expression in <i>Drosophila</i> models of Parkinson's disease; FASEB JOURNAL	2020	Excluded	Wrong population
García-Villalba R and Tomás-Barberán FA and Iglesias-Aguirre CE and Giménez-Bastida JA and González-Sarrías A and Selma MV and Espín JC; Ellagitannins, urolithins, and neuroprotection: Human evidence and the possible link to the gut microbiota.; Molecular aspects of medicine	2023	Excluded	Wrong publication type
Angelopoulou, E. and Paudel, Y.N. and Papageorgiou, S.G. and Piperi, C.;	2022	Excluded	Wrong publication type

Elucidating the Beneficial Effects of Ginger (Zingiber officinale Roscoe) in Parkinson's Disease; ACS Pharmacology and Translational Science			
Yao, L. and Liu, W. and Bashir, M. and Nisar, M.F. and Wan, C.C.; Eriocitrin: A review of pharmacological effects; Biomedicine and Pharmacotherapy	2022	Excluded	Wrong publication type
Charde, V. and Kumar, V. and Dane, G. and Gandhi, Y. and Soni, H. and Jagtap, C. and Mishra, S.K. and Shakya, S.K. and Singh, A. and Singh, R. and Sharma, B.S. and Khanduri, S. and Srikanth, N. and Acharya, R. and Webster, T.J.; Establishment of the mechanism of purification and levigation of green chemistry-assisted biocomposites of red ochre (Gairika): synthesis, characterization, and antibacterial, prebiotic, antioxidant, and antacid activities of the traditional Ayurvedic medicine Laghu Sutashekhara Rasa; Frontiers in Chemistry	2023	Excluded	Wrong publication type
Lekchand Dasriya V and Samtiya M and Dhewa T and Puniya M and Kumar S and Ranveer S and Chaudhary V and Vij S and Behare P and Singh N and Aluko RE and Puniya AK; Etiology and management of Alzheimer's disease: Potential role of gut microbiota modulation with probiotics supplementation.; Journal of food biochemistry	2022	Excluded	Wrong publication type
Kou, J. and Kang, H. and Hu, L. and Wang, D. and Wang, S. and Wang, Q. and Yang, Z.; Evaluation of improvement of cognitive impairment in older adults with probiotic supplementation: A systematic review and meta-analysis; Geriatric Nursing	2023	Excluded	Wrong publication type
Giménez-Bastida, J.A. and Ávila-Gálvez, M.Á. and Espín, J.C. and González-Sarrías, A.; Evidence for health properties of pomegranate juices and extracts beyond nutrition: A critical	2021	Excluded	Wrong publication type

systematic review of human studies; Trends in Food Science and Technology			
Matei, B. and Winters-Stone, K.M. and Raber, J.; Examining the Mechanisms behind Exercise's Multifaceted Impacts on Body Composition, Cognition, and the Gut Microbiome in Cancer Survivors: Exploring the Links to Oxidative Stress and Inflammation; Antioxidants	2023	Excluded	Wrong population
Zhong, L. and Cai, B. and Wang, Q. and Li, X. and Xu, W. and Chen, T.; Exploring the Neuroprotective Mechanism of Curcumin Inhibition of Intestinal Inflammation against Parkinson's Disease Based on the Gut-Brain Axis; Pharmaceuticals	2023	Excluded	Wrong population
Scorza, C. and Goncalves, V. and Finsterer, J. and Scorza, F. and Fonseca, F.; Exploring the Prospective Role of Propolis in Modifying Aging Hallmarks; Cells	2024	Excluded	Wrong publication type
Ghosh A and Muley A and Bhat S and Ainapure A; Exploring the Renoprotective Potential of Bioactive Nutraceuticals in Chronic Kidney Disease Progression: A Narrative Review.; Cureus	2024	Excluded	Wrong publication type
Sandhu, K.V. and Sherwin, E. and Schellekens, H. and Stanton, C. and Dinan, T.G. and Cryan, J.F.; Feeding the microbiota-gut-brain axis: diet, microbiome, and neuropsychiatry; Translational Research	2017	Excluded	Wrong publication type
Gopnar, V.V. and Rakshit, D. and Bandakinda, M. and Kulhari, U. and Sahu, B.D. and Mishra, A.; Fisetin attenuates arsenic and fluoride subacute co-exposure induced neurotoxicity via regulating TNF-α mediated activation of NLRP3 inflammasome; NeuroToxicology	2023	Excluded	Wrong outcome
Magni, G. and Riboldi, B. and Petroni, K. and Ceruti, S.; Flavonoids bridging the gut and the brain: Intestinal metabolic fate, and direct or indirect effects of natural supporters against	2022	Excluded	Wrong publication type

neuroinflammation and neurodegeneration; Biochemical Pharmacology			
Xia, C.-X. and Gao, A.X. and Zhu, Y. and Dong, T.T. and Tsim, K.W.; Flavonoids from Seabuckthorn (Hippophae rhamnoides L.) restore CUMS-induced depressive disorder and regulate the gut microbiota in mice; Food and Function	2023	Excluded	Wrong population
Açar, Y. and Ağagündüz, D. and De Cicco, P. and Capasso, R.; Flavonoids: Their putative neurologic roles, epigenetic changes, and gut microbiota alterations in Parkinson's disease; Biomedicine and Pharmacotherapy	2023	Excluded	Wrong publication type
Avila-Nava, A. and Noriega, L.G. and Tovar, A.R. and Granados, O. and Perez-Cruz, C. and Pedraza-Chaverri, J. and Torres, N.; Food combination based on a pre-hispanic Mexican diet decreases metabolic and cognitive abnormalities and gut microbiota dysbiosis caused by a sucrose-enriched high-fat diet in rats; Molecular Nutrition and Food Research	2017	Excluded	Wrong population
Anand, A. and Khurana, N. and Kumar, R. and Sharma, N.; Food for the mind: The journey of probiotics from foods to anti-Alzheimer's disease therapeutics; Food Bioscience	2023	Excluded	Wrong publication type
Jin, Y. and Kim, T. and Kang, H.; Forced treadmill running modifies gut microbiota with alleviations of cognitive impairment and Alzheimer's disease pathology in 3xTg-AD mice; Physiology and Behavior	2023	Excluded	Wrong population
Domínguez-López I and López-Yerena A and Vallverdú-Queralt A and Pallàs M and Lamuela-Raventós RM and Pérez M; From the gut to the brain: the long journey of phenolic compounds with neurocognitive effects.; Nutrition reviews	2024	Excluded	Wrong publication type
Li, L. and Chen, B. and Zhu, R. and Li, R. and Tian, Y. and Liu, C. and Jia, Q. and Wang, L. and Tang, J. and Zhao, D. and Mo, F. and Liu, Y. and Li, Y. and	2019	Excluded	Wrong population

Orekhov, A.N. and Brömme, D. and Zhang, D. and Gao, S.; Fructus Ligustri Lucidi preserves bone quality through the regulation of gut microbiota diversity, oxidative stress, TMAO and Sirt6 levels in aging mice; Aging			
Wang, Y. and Wang, Q. and Duan, L. and Li, X. and Yang, W. and Huang, T. and Kong, M. and Guan, F. and Ma, S.; Fucoidan ameliorates LPS-induced neuronal cell damage and cognitive impairment in mice; International Journal of Biological Macromolecules	2022	Excluded	Wrong population
Yang, M. and Xuan, Z. and Wang, Q. and Yan, S. and Zhou, D. and Naman, C.B. and Zhang, J. and He, S. and Yan, X. and Cui, W.; Fucoxanthin has potential for therapeutic efficacy in neurodegenerative disorders by acting on multiple targets; Nutritional Neuroscience	2022	Excluded	Wrong publication type
Liu, M. and Li, W. and Chen, Y. and Wan, X. and Wang, J.; Fucoxanthin: A promising compound for human inflammation-related diseases; Life Sciences	2020	Excluded	Wrong publication type
Atlante, A. and Amadoro, G. and Bobba, A. and Latina, V.; Functional foods: An approach to modulate molecular mechanisms of alzheimer's disease; Cells	2020	Excluded	Wrong publication type
Berlamont, H. and Bruggeman, A. and Bauwens, E. and Vandendriessche, C. and Clarebout, E. and Xie, J. and De Bruyckere, S. and Van Imschoot, G. and Van Wonterghem, E. and Ducatelle, R. and Santens, P. and Smet, A. and Haesebrouck, F. and Vandenbroucke, R.E.; Gastric helicobacter suis infection partially protects against neurotoxicity in a 6-ohda parkinson's disease mouse model; International Journal of Molecular Sciences	2021	Excluded	Wrong population
Huang, Y.-J. and Choong, L.-X.C. and Panyod, S. and Lin, Y.-E. and Huang, H.-S. and Lu, K.-H. and Wu, W.-K. and Sheen, L.-Y.; Gastrodia elata Blume	2021	Excluded	Wrong population

water extract modulates neurotransmitters and alters the gut microbiota in a mild social defeat stress-induced depression mouse model; Phytotherapy Research			
Naeimi, M. and Gorji, N. and Memariani, Z. and Moeini, R. and Kamalinejad, M. and Kolangi, F.; Gastroprotective herbs for headache management in Persian medicine: A comprehensive review; Journal of Integrative Medicine	2020	Excluded	Wrong publication type
Clemente-Suárez, V.J. and Beltrán-Velasco, A.I. and Redondo-Flórez, L. and Martín-Rodríguez, A. and Tornero-Aguilera, J.F.; Global Impacts of Western Diet and Its Effects on Metabolism and Health: A Narrative Review; Nutrients	2023	Excluded	Wrong publication type
Izumi, Y. and O'Dell, K.A. and Zorumski, C.F.; Glyphosate as a direct or indirect activator of pro-inflammatory signaling and cognitive impairment; Neural Regeneration Research	2024	Excluded	Wrong publication type
Berding K and Carbia C and Cryan JF; Going with the grain: Fiber, cognition, and the microbiota-gut-brain-axis.; Experimental biology and medicine (Maywood, N.J.)	2021	Excluded	Wrong publication type
Hsu, Y.-C. and Huang, Y.-Y. and Tsai, S.-Y. and Kuo, Y.-W. and Lin, J.-H. and Ho, H.-H. and Chen, J.-F. and Hsia, K.-C. and Sun, Y.; Efficacy of Probiotic Supplements on Brain-Derived Neurotrophic Factor, Inflammatory Biomarkers, Oxidative Stress and Cognitive Function in Patients with Alzheimer's Dementia: A 12-Week Randomized, Double-Blind Active-Controlled Study; Nutrients	2024	Included	RCT
Li, S. and Zhao, X. and Lin, F. and Ni, X. and Liu, X. and Kong, C. and Yao, X. and Mo, Y. and Dai, Q. and Wang, J.; Gut Flora Mediates the Rapid Tolerance of Electroacupuncture on Ischemic Stroke by Activating Melatonin Receptor	2022	Excluded	Wrong population

through Regulating Indole-3-Propionic Acid; American Journal of Chinese Medicine			
Bauer, K.C. and York, E.M. and Cirstea, M.S. and Radisavljevic, N. and Petersen, C. and Huus, K.E. and Brown, E.M. and Bozorgmehr, T. and Berdún, R. and Bernier, L.-P. and Lee, A.H.Y. and Woodward, S.E. and Krekhno, Z. and Han, J. and Hancock, R.E.W. and Ayala, V. and MacVicar, B.A. and Finlay, B.B.; Gut microbes shape microglia and cognitive function during malnutrition; GLIA	2022	Excluded	Wrong publication type
Gade, A. and Kumar, M.S.; Gut microbial metabolites of dietary polyphenols and their potential role in human health and diseases; Journal of Physiology and Biochemistry	2023	Excluded	Wrong publication type
Guha, L. and Agnihotri, T.G. and Jain, A. and Kumar, H.; Gut microbiota and traumatic central nervous system injuries: Insights into pathophysiology and therapeutic approaches; Life Sciences	2023	Excluded	Wrong publication type
Zhang, M. and Mo, R. and Wang, H. and Liu, T. and Zhang, G. and Wu, Y.; Grape seed proanthocyanidin improves intestinal inflammation in canine through regulating gut microbiota and bile acid compositions; FASEB Journal	2023	Included	Not a RCT
Zhang, H. and Xu, J. and Wu, Q. and Fang, H. and Shao, X. and Ouyang, X. and He, Z. and Deng, Y. and Chen, C.; Gut Microbiota Mediates the Susceptibility of Mice to Sepsis-Associated Encephalopathy by Butyric Acid; Journal of Inflammation Research	2022	Excluded	Wrong population
Ortega, M.A. and Alvarez-Mon, M.A. and García-Montero, C. and Fraile-Martinez, O. and Guijarro, L.G. and Lahera, G. and Monserrat, J. and Valls, P. and Mora, F. and Rodríguez-Jiménez, R. and Quintero, J. and Álvarez-Mon, M.; Gut Microbiota Metabolites in Major Depressive Disorder—Deep Insights into Their Pathophysiological Role and	2022	Excluded	Wrong publication type

Potential Translational Applications; Metabolites			
Marques C and Fernandes I and Meireles M and Faria A and Spencer JPE and Mateus N and Calhau C; Gut microbiota modulation accounts for the neuroprotective properties of anthocyanins.; Scientific reports	2018	Excluded	Wrong population
Santino A and Scarano A and De Santis S and De Benedictis M and Giovinazzo G and Chieppa M; Gut Microbiota Modulation and Anti-Inflammatory Properties of Dietary Polyphenols in IBD: New and Consolidated Perspectives.; Current pharmaceutical design	2017	Excluded	Wrong publication type
Ticinesi, A and Tana, C and Nouvenne, A and Prati, B and Lauretani, F and Meschi, T; Gut microbiota, cognitive frailty and dementia in older individuals: a systematic review; CLINICAL INTERVENTIONS IN AGING	2018	Excluded	Wrong publication type
He, Y. and Li, B. and Sun, D. and Chen, S.; Gut microbiota: Implications in Alzheimer's disease; Journal of Clinical Medicine	2020	Excluded	Wrong publication type
Rudzki, L. and Stone, T.W. and Maes, M. and Misiak, B. and Samochowiec, J. and Szulc, A.; Gut microbiota-derived vitamins – underrated powers of a multipotent ally in psychiatric health and disease; Progress in Neuro-Psychopharmacology and Biological Psychiatry	2021	Excluded	Wrong publication type
Riegelman, E. and Xue, K.S. and Wang, J.-S. and Tang, L.; Gut–Brain Axis in Focus: Polyphenols, Microbiota, and Their Influence on α-Synuclein in Parkinson's Disease; Nutrients	2024	Excluded	Wrong publication type
Zhang, X. and Song, X. and Hu, X. and Chen, F. and Ma, C.; Health benefits of proanthocyanidins linking with gastrointestinal modulation: An updated review; Food Chemistry	2023	Excluded	Wrong publication type

Rossi I and Mignogna C and Del Rio D and Mena P; Health effects of 100% fruit and vegetable juices: evidence from human subject intervention studies.; Nutrition research reviews	2024	Excluded	Wrong publication type
Kolb, H. and Kempf, K. and Martin, S.; Health effects of coffee: Mechanism unraveled?; Nutrients	2020	Excluded	Wrong publication type
Calder, P.C. and Bosco, N. and Bourdet-Sicard, R. and Capuron, L. and Delzenne, N. and Doré, J. and Franceschi, C. and Lehtinen, M.J. and Recker, T. and Salvioli, S. and Visioli, F.; Health relevance of the modification of low grade inflammation in ageing (inflammageing) and the role of nutrition; Ageing Research Reviews	2017	Excluded	Wrong publication type
Guan, Y. and Tang, G. and Li, L. and Shu, J. and Zhao, Y. and Huang, L. and Tang, J.; Herbal medicine and gut microbiota: exploring untapped therapeutic potential in neurodegenerative disease management; Archives of Pharmacal Research	2024	Excluded	Wrong publication type
Guan, Y. and Shi, D. and Wang, S. and Sun, Y. and Song, W. and Liu, S. and Wang, C.; Hericium coralloides Ameliorates Alzheimer's Disease Pathologies and Cognitive Disorders by Activating Nrf2 Signaling and Regulating Gut Microbiota; Nutrients	2023	Excluded	Wrong publication type
Zhao, L. and Zhang, C. and Cao, G. and Dong, X. and Li, D. and Jiang, L.; Higher Circulating Trimethylamine N-oxide Sensitizes Sevoflurane-Induced Cognitive Dysfunction in Aged Rats Probably by Downregulating Hippocampal Methionine Sulfoxide Reductase A; Neurochemical Research	2019	Excluded	Wrong population
Scuto M and Rampulla F and Reali GM and Spanò SM and Trovato Salinaro A and Calabrese V; Hormetic Nutrition and Redox Regulation in Gut-Brain Axis Disorders.; Antioxidants (Basel, Switzerland)	2024	Excluded	Wrong publication type

Osakabe, N and Fushimi, T and Fujii, Y; Hormetic response to B-type procyanidin ingestion involves stress-related neuromodulation via the gut-brain axis: Preclinical and clinical observations; FRONTIERS IN NUTRITION	2022	Excluded	Wrong publication type
Bulut, O. and Kilic, G. and Domínguez-Andrés, J.; Immune Memory in Aging: a Wide Perspective Covering Microbiota, Brain, Metabolism, and Epigenetics; Clinical Reviews in Allergy and Immunology	2022	Excluded	Wrong publication type
Girolamo, F. and Coppola, C. and Ribatti, D.; Immunoregulatory effect of mast cells influenced by microbes in neurodegenerative diseases; Brain, Behavior, and Immunity	2017	Excluded	Wrong population
Hegde, P.S. and Agni, M.B. and Rai, P. and Mohana Kumar, B. and Damodara Gowda, K.M.; Impact of carotenoids on gut microbiome: Implications in human health and disease; Journal of Applied and Natural Science	2022	Excluded	Wrong publication type
Yao, X. and Wang, A. and Gao, B. and Xue, Y. and Zhao, X. and Wang, X. and Wang, R. and Zhuang, M. and Wei, C. and Cheng, W. and Zhang, J. and Strappe, P. and Zhou, Z.; Impact of de-branched starch molecules and fatty acid complexes on the attenuation of ageing-induced cognitive impairment; Journal of the Science of Food and Agriculture	2023	Excluded	Wrong publication type
Ahmed, S. and Busetti, A. and Fotiadou, P. and Vincy Jose, N. and Reid, S. and Georgieva, M. and Brown, S. and Dunbar, H. and Beurket-Ascencio, G. and Delday, M.I. and Ettorre, A. and Mulder, I.E.; In vitro Characterization of Gut Microbiota-Derived Bacterial Strains With Neuroprotective Properties; Frontiers in Cellular Neuroscience	2019	Excluded	Wrong population
Michael, D.R. and Davies, T.S. and Loxley, K.E. and Allen, M.D. and Good, M.A. and Hughes, T.R. and Plummer,	2019	Excluded	Wrong population

S.F.; In vitro neuroprotective activities of two distinct probiotic consortia; Beneficial Microbes			
Pappolla, M.A. and Perry, G. and Fang, X. and Zagorski, M. and Sambamurti, K. and Poeggeler, B.; Indoles as essential mediators in the gut-brain axis. Their role in Alzheimer's disease; Neurobiology of Disease	2021	Excluded	Wrong publication type
Ildarabadi, A. and Ali, S.N.M.M. and Rahmani, F. and Mosavari, N. and Pourbakhtyaran, E. and Rezaei, N.; Inflammation and oxidative stress in epileptic children: from molecular mechanisms to clinical application of ketogenic diet; Reviews in the Neurosciences	2024	Excluded	Wrong publication type
Cirulli, F. and De Simone, R. and Musillo, C. and Ajmone-Cat, M.A. and Berry, A.; Inflammatory Signatures of Maternal Obesity as Risk Factors for Neurodevelopmental Disorders: Role of Maternal Microbiota and Nutritional Intervention Strategies; Nutrients	2022	Excluded	Wrong publication type
Heidari M and Khodadadi Jokar Y and Madani S and Shahi S and Shahi MS and Goli M; Influence of Food Type on Human Psychological-Behavioral Responses and Crime Reduction.; Nutrients	2023	Excluded	Wrong publication type
Xu, L. and Zeng, X. and Liu, Y. and Wu, Z. and Zheng, X. and Zhang, X.; Inhibitory effect of Dendrobium officinale polysaccharide on oxidative damage of glial cells in aging mice by regulating gut microbiota; International Journal of Biological Macromolecules	2023	Excluded	Wrong population
Wang, W. and Ige, O.O. and Ding, Y. and He, M. and Long, P. and Wang, S. and Zhang, Y. and Wen, X.; Insights into the potential benefits of triphala polyphenols toward the promotion of resilience against stress-induced depression and cognitive impairment; Current Research in Food Science	2023	Excluded	Wrong publication type
Das, T.K. and Ganesh, B.P.; Interlink between the gut microbiota and	2023	Excluded	Wrong publication type

inflammation in the context of oxidative stress in Alzheimer's disease progression; Gut Microbes			
Salami, M.; Interplay of Good Bacteria and Central Nervous System: Cognitive Aspects and Mechanistic Considerations; Frontiers in Neuroscience	2021	Excluded	Wrong publication type
Shandilya, S. and Kumar, S. and Kumar Jha, N. and Kumar Kesari, K. and Ruokolainen, J.; Interplay of gut microbiota and oxidative stress: Perspective on neurodegeneration and neuroprotection; Journal of Advanced Research	2022	Excluded	Wrong publication type
Jamieson, P.E.; Smart, E.B.; Bouranis, J.A.; Choi, J.; Danczak, R.E.; Wong, C.P.; Paraiso, I.L.; Maier, C.S.; Ho, E.; Sharpton, T.J.; et al. ; Gut Enterotype-Dependent Modulation of Gut Microbiota and Their Metabolism in Response to Xanthohumol Supplementation in Healthy Adults; Gut Microbes	2024	Included	RCT
Horasan Sagbasan B and Williams CM and Bell L and Barfoot KL and Poveda C and Walton GE; Inulin and Freeze-Dried Blueberry Intervention Lead to Changes in the Microbiota and Metabolites within In Vitro Studies and in Cognitive Function within a Small Pilot Trial on Healthy Children.; Microorganisms	2024	Excluded	Wrong population
Balasubramanian, R. and Bazaz, M.R. and Pasam, T. and Sharief, N. and Velip, L. and Samanthula, G. and Dandekar, M.P.; Involvement of Microbiome Gut–Brain Axis in Neuroprotective Effect of Quercetin in Mouse Model of Repeated Mild Traumatic Brain Injury; NeuroMolecular Medicine	2023	Excluded	Wrong population
Patil, T. and Patil, S. and Patil, A. and Patil, S.; Is berberine superior to metformin in management of diabetes mellitus and its complications?; International Journal of Pharmacognosy and Phytochemical Research	2015	Excluded	Wrong publication type

Zakharova, I.N. and Berezhnaya, I.V.; Is it possible to prevent respiratory pathology?; Meditsinskiy Sovet	2020	Excluded	Wrong publication type
Wang, J. and Pan, J. and Zou, J. and Shi, Y. and Guo, D. and Zhai, B. and Zhao, C. and Luan, F. and Zhang, X. and Sun, J.; Isolation, structures, bioactivities, and utilizations of polysaccharides from Dictyophora species: A review; International Journal of Biological Macromolecules	2024	Excluded	Wrong publication type
Tao, Y. and Leng, S.X. and Zhang, H.; Ketogenic Diet: An Effective Treatment Approach for Neurodegenerative Diseases; Current Neuropharmacology	2022	Excluded	Wrong outcome
Cai, Y. and Dong, Y. and Han, M. and Jin, M. and Liu, H. and Gai, Z. and Zou, K.; Lacticaseibacillus paracasei LC86 mitigates age-related muscle wasting and cognitive impairment in SAMP8 mice through gut microbiota modulation and the regulation of serum inflammatory factors; Frontiers in Nutrition	2024	Excluded	Wrong population
Beltrán-Velasco, A.I. and Reiriz, M. and Uceda, S. and Echeverry-Alzate, V.; Lactiplantibacillus (Lactobacillus) plantarum as a Complementary Treatment to Improve Symptomatology in Neurodegenerative Disease: A Systematic Review of Open Access Literature; International Journal of Molecular Sciences	2024	Excluded	Wrong publication type
Cheng, L.-H. and Chou, P.-Y. and Hou, A.-T. and Huang, C.-L. and Shiu, W.-L. and Wang, S.; Lactobacillus paracasei PS23 improves cognitive deficits via modulating the hippocampal gene expression and the gut microbiota in d-galactose-induced aging mice; Food and Function	2022	Excluded	Wrong population
Shi, R. and Ye, J. and Fan, H. and Hu, X. and Wu, X. and Wang, D. and Zhao, B. and Dai, X. and Liu, X.; Lactobacillus plantarum LLY-606 Supplementation Ameliorates the Cognitive Impairment of Natural Aging in Mice: The Potential	2024	Excluded	Wrong population

Role of Gut Microbiota Homeostasis; Journal of Agricultural and Food Chemistry			
Du, L. and Chen, J. and Yan, J. and Xie, H. and Wang, L. and Wang, R. and Han, X. and Wang, Y.; Lingguizhugan decoction ameliorates cognitive impairment in AD-like mice by influencing the microbiome–gut–brain axis mediated by SCFAs; Phytomedicine	2024	Excluded	Wrong population
Zhang, Y. and Wang, Y. and Zhou, Z. and Yang, Y. and Zhao, J. and Kang, X. and Li, Z. and Shen, X. and He, F. and Cheng, R.; Live and Heat-Inactivated Streptococcus thermophilus MN-ZLW-002 Mediate the Gut–Brain Axis, Alleviating Cognitive Dysfunction in APP/PS1 Mice; Nutrients	2024	Excluded	Wrong population
Wang, J. and Shen, Y. and Li, L. and Li, L. and Zhang, J. and Li, M. and Qiu, F.; Lycopene attenuates D-galactose-induced memory and behavioral deficits by mediating microbiota-SCFAs-gut-brain axis balance in female CD-1 mice; Journal of Nutritional Biochemistry	2025	Excluded	Wrong population
Fakhri, S. and Yarmohammadi, A. and Yarmohammadi, M. and Farzaei, M.H. and Echeverria, J.; Marine Natural Products: Promising Candidates in the Modulation of Gut-Brain Axis towards Neuroprotection; Marine Drugs	2021	Excluded	Wrong publication type
Yousef, O. and De Souza, S.; Meals and movies: making our microbiota merry; Medical Journal of Australia	2022	Excluded	Wrong publication type
Dobielska, M. and Bartosik, N.K. and Zyzik, K.A. and Kowalczyk, E. and Karbownik, M.S.; Mechanisms of Cognitive Impairment in Depression. May Probiotics Help?; Frontiers in Psychiatry	2022	Excluded	Wrong publication type
Pferschy-Wenzig, E.-M. and Pausan, M.R. and Ardjomand-Woelkart, K. and Röck, S. and Ammar, R.M. and Kelber, O. and Moissl-Eichinger, C. and Bauer, R.; Medicinal Plants and Their Impact	2022	Excluded	Wrong publication type

on the Gut Microbiome in Mental Health: A Systematic Review; Nutrients			
Gantenbein, K.V. and Kanaka-Gantenbein, C.; Mediterranean diet as an antioxidant: The impact on metabolic health and overall wellbeing; Nutrients	2021	Excluded	Wrong publication type
Xie, L. and Wu, H. and Huang, X. and Yu, T.; Melatonin, a natural antioxidant therapy in spinal cord injury; Frontiers in Cell and Developmental Biology	2023	Excluded	Wrong publication type
Milošević, M. and Arsić, A. and Cvetković, Z. and Vučić, V.; Memorable Food: Fighting Age-Related Neurodegeneration by Precision Nutrition; Frontiers in Nutrition	2021	Excluded	Wrong publication type
Wang, LF and Ren, B and Hui, Y and Chu, CQ and Zhao, ZT and Zhang, YY and Zhao, BT and Shi, RJ and Ren, JL and Dai, XS and Liu, ZG and Liu, XB; Methionine Restriction Regulates Cognitive Function in High-Fat Diet-Fed Mice: Roles of Diurnal Rhythms of SCFAs Producing- and Inflammation-Related Microbes; MOLECULAR NUTRITION & FOOD RESEARCH	2020	Excluded	Wrong publication type
Connell, E. and Le Gall, G. and Pontifex, M.G. and Sami, S. and Cryan, J.F. and Clarke, G. and Müller, M. and Vauzour, D.; Microbial-derived metabolites as a risk factor of age-related cognitive decline and dementia; Molecular Neurodegeneration	2022	Excluded	Wrong publication type
Onaolapo, A.Y. and Ojo, F.O. and Olofinnade, A.T. and Falade, J. and Lawal, I.A. and Onaolapo, O.J.; Microbiome-Based Therapies in Parkinson’s Disease: Can Tuning the Microbiota Become a Viable Therapeutic Strategy?; CNS and Neurological Disorders - Drug Targets	2023	Excluded	Wrong publication type
Ağagündüz, D. and Kocaadam-Bozkurt, B. and Bozkurt, O. and Sharma, H. and Esposito, R. and Özoğul, F. and Capasso, R.; Microbiota alteration and modulation in Alzheimer's disease by gerobiotics: The gut-health axis for a	2022	Excluded	Wrong publication type

good mind; Biomedicine and Pharmacotherapy			
Singh, J. and Singh, A. and Biswal, S. and Zomuansangi, R. and Lalbiaktluangi, C. and Singh, B.P. and Singh, P.K. and Vellingiri, B. and Iyer, M. and Ram, H. and Udey, B. and Yadav, M.K.; Microbiota-brain axis: Exploring the role of gut microbiota in psychiatric disorders - A comprehensive review; Asian Journal of Psychiatry	2024	Excluded	Wrong publication type
Li, J. and Zhang, F. and Zhao, L. and Dong, C.; Microbiota-gut-brain axis and related therapeutics in Alzheimer's disease: Prospects for multitherapy and inflammation control; Reviews in the Neurosciences	2023	Excluded	Wrong publication type
Lu, Y. and Yu, X. and Wang, Z. and Kong, L. and Jiang, Z. and Shang, R. and Zhong, X. and Lv, S. and Zhang, G. and Gao, H. and Yang, N.; Microbiota–gut–brain axis: Natural antidepressants molecular mechanism; Phytomedicine	2024	Excluded	Wrong publication type
Faradina A and Tinkov AA and Skalny AV and Chang JS; Micronutrient (iron, selenium, vitamin D) supplementation and the gut microbiome.; Current opinion in clinical nutrition and metabolic care	2024	Excluded	Wrong publication type
Albuquerque Pereira, M.F. and Morais de Ávila, L.G. and Ávila Alpino, G.C. and dos Santos Cruz, B.C. and Almeida, L.F. and Macedo Simões, J. and Ladeira Bernardes, A. and Xisto Campos, I. and de Oliveira Barros Ribon, A. and de Oliveira Mendes, T.A. and Gouveia Peluzio, M.C.; Milk kefir alters fecal microbiota impacting gut and brain health in mice; Applied Microbiology and Biotechnology	2023	Excluded	Wrong population
Kulchavenia, E.V.; Minerals contributing to human health and well-being; Clinical Review for General Practice	2021	Excluded	Wrong publication type
Heberden, C.; Modulating adult neurogenesis through dietary	2016	Excluded	Wrong publication type

interventions; Nutrition Research Reviews			
Wang, X. and Yang, C. and Yang, L. and Zhang, Y.; Modulating the gut microbiota ameliorates spontaneous seizures and cognitive deficits in rats with kainic acid-induced status epilepticus by inhibiting inflammation and oxidative stress; Frontiers in Nutrition	2022	Excluded	Wrong publication type
Liu, M. and Zhong, P.; Modulating the Gut Microbiota as a Therapeutic Intervention for Alzheimer's Disease; Indian Journal of Microbiology	2022	Excluded	Wrong publication type
Munawar, N. and Ahmad, A. and Anwar, M.A. and Muhammad, K.; Modulation of Gut Microbial Diversity through Non-Pharmaceutical Approaches to Treat Schizophrenia; International Journal of Molecular Sciences	2022	Excluded	Wrong publication type
Yousof, S.M. and Alghamdi, B.S. and Alqurashi, T. and Alam, M.Z. and Tash, R. and Tanvir, I. and Kaddam, L.A.; Modulation of Gut Microbiome Community Mitigates Multiple Sclerosis in a Mouse Model: The Promising Role of Palmaria palmata Alga as a Prebiotic; Pharmaceuticals	2023	Excluded	Wrong population
Tripathi AK and Ray AK and Mishra SK and Bishen SM and Mishra H and Khurana A; Molecular and Therapeutic Insights of Alpha-Lipoic Acid as a Potential Molecule for Disease Prevention.; Revista brasileira de farmacognosia : orgao oficial da Sociedade Brasileira de Farmacognosia	2023	Excluded	Wrong publication type
Luqman, A. and He, M. and Hassan, A. and Ullah, M. and Zhang, L. and Rashid Khan, M. and Din, A.U. and Ullah, K. and Wang, W. and Wang, G.; Mood and microbes: a comprehensive review of intestinal microbiota's impact on depression; Frontiers in Psychiatry	2024	Excluded	Wrong publication type

Ishola, I.O. and Awogbindin, I.O. and Olubodun-Obadun, T.G. and Oluwafemi, O.A. and Onuelu, J.E. and Adeyemi, O.O.; Morin ameliorates rotenone-induced Parkinson disease in mice through antioxidation and anti-neuroinflammation: gut-brain axis involvement; Brain Research	2022	Excluded	Wrong population
Song, Z. and Ho, C.-T. and Zhang, X.; Gut Microbiota Mediate the Neuroprotective Effect of Oolong Tea Polyphenols in Cognitive Impairment Induced by Circadian Rhythm Disorder; Journal of Agricultural and Food Chemistry	2024	Included	Not a RCT
Pluta, R. and Januszewski, S. and Ułamek-kozioł, M.; Mutual two-way interactions of curcumin and gut microbiota; International Journal of Molecular Sciences	2020	Excluded	Wrong publication type
Wu, J. and Cui, X. and Ke, P.C. and Mortimer, M. and Wang, X. and Bao, L. and Chen, C.; Nanomaterials as novel agents for amelioration of Parkinson's disease; Nano Today	2021	Excluded	Wrong publication type
Dou, Z. and Rong, X. and Zhao, E. and Zhang, L. and Lv, Y.; Neuroprotection of Resveratrol Against Focal Cerebral Ischemia/Reperfusion Injury in Mice Through a Mechanism Targeting Gut-Brain Axis; Cellular and Molecular Neurobiology	2019	Excluded	Wrong population
Deng, Y. and Li, Q. and Song, J. and Guo, R. and Ma, T. and Liu, Z. and Liu, Q.; Intervention effects of low-molecular-weight chondroitin sulfate from the nasal cartilage of yellow cattle on lipopolysaccharide-induced behavioral disorders: regulation of the microbiome-gut-brain axis; Frontiers in Nutrition	2024	Included	Animals
Zhang, X. and Guo, F. and Cao, D. and Yan, Y. and Zhang, N. and Zhang, K. and Li, X. and Kumar, P.; Neuroprotective Effect of Ponicidin Alleviating the Diabetic Cognitive Impairment: Regulation of Gut	2023	Excluded	Wrong publication type

Microbiota; Applied Biochemistry and Biotechnology			
Park, J. and Lee, J. and Yeom, Z. and Heo, D. and Lim, Y.-H.; Neuroprotective effect of Ruminococcus albus on oxidatively stressed SH-SY5Y cells and animals; Scientific Reports	2017	Excluded	Wrong population
Pérez Visñuk, D. and LeBlanc, J.G. and de Moreno de LeBlanc, A.; Neuroprotective Effects Exerted by a Combination of Selected Lactic Acid Bacteria in a Mouse Parkinsonism Model under Levodopa-Benserazide Treatment; Neurochemical Research	2024	Excluded	Wrong population
Li, T. and Chu, C. and Yu, L. and Zhai, Q. and Wang, S. and Zhao, J. and Zhang, H. and Chen, W. and Tian, F.; Neuroprotective Effects of Bifidobacterium breve CCFM1067 in MPTP-Induced Mouse Models of Parkinson's Disease; Nutrients	2022	Excluded	Wrong population
Cheon, M.-J. and Lee, N.-K. and Paik, H.-D.; Neuroprotective Effects of Heat-Killed Lactobacillus plantarum 200655 Isolated from Kimchi Against Oxidative Stress; Probiotics and Antimicrobial Proteins	2021	Excluded	Wrong population
Bock, H.-J. and Lee, N.-K. and Paik, H.-D.; Neuroprotective Effects of Heat-Killed Levilactobacillus brevis KU15152 on H2O2Induced Oxidative Stress; Journal of Microbiology and Biotechnology	2023	Excluded	Wrong population
Liaqat, H. and Parveen, A. and Kim, S.Y.; Neuroprotective Natural Products' Regulatory Effects on Depression via Gut–Brain Axis Targeting Tryptophan; Nutrients	2022	Excluded	Wrong publication type
Tian, E. and Sharma, G. and Dai, C.; Neuroprotective Properties of Berberine: Molecular Mechanisms and Clinical Implications; Antioxidants	2023	Excluded	Wrong publication type
Yildiran, H. and Macit, M.S. and Özata Uyar, G.; New approach to peripheral nerve injury: nutritional therapy; Nutritional Neuroscience	2020	Excluded	Wrong publication type

Fisette A and Sergi D and Breton-Morin A and Descôteaux S and Martinoli MG; New Insights on the Role of Bioactive Food Derivatives in Neurodegeneration and Neuroprotection.; Current pharmaceutical design	2022	Excluded	Wrong publication type
Di Meo, F. and Donato, S. and Di Pardo, A. and Maglione, V. and Filosa, S. and Crispi, S.; New therapeutic drugs from bioactive natural molecules: The role of gut microbiota metabolism in neurodegenerative diseases; Current Drug Metabolism	2018	Excluded	Wrong publication type
Mehta, R. and Kuhad, A. and Bhandari, R.; Nitric oxide pathway as a plausible therapeutic target in autism spectrum disorders; Expert Opinion on Therapeutic Targets	2022	Excluded	Wrong outcome
Sánchez-Tapia M and Aguilar-López M and Pérez-Cruz C and Pichardo-Ontiveros E and Wang M and Donovan SM and Tovar AR and Torres N; Nopal (Opuntia ficus indica) protects from metabolic endotoxemia by modifying gut microbiota in obese rats fed high fat/sucrose diet.; Scientific reports	2017	Excluded	Wrong population
Jafari, R.S. and Behrouz, V.; Nordic diet and its benefits in neurological function: a systematic review of observational and intervention studies; Frontiers in Nutrition	2023	Excluded	Wrong publication type
Premachandran, K and Alphonse, CRW and Soundharapandiyan, N; Nourishing the Cognition with Millets: A Comprehensive Review of Their Nutritional Impact and Potential as Cognitive Enhancers; MOLECULAR NUTRITION & FOOD RESEARCH	2023	Excluded	Wrong publication type
Wu, S. and Wu, Q. and Wang, J. and Li, Y. and Chen, B. and Zhu, Z. and Huang, R. and Chen, M. and Huang, A. and Xie, Y. and Jiao, C. and Ding, Y.; Novel Selenium Peptides Obtained from Selenium-Enriched Cordyceps militaris Alleviate Neuroinflammation and Gut Microbiota Dysbacteriosis in LPS-	2022	Excluded	Wrong population

Injured Mice; Journal of Agricultural and Food Chemistry			
Sadovnikova, I.S. and Gureev, A.P. and Ignatyeva, D.A. and Gryaznova, M.V. and Chernyshova, E.V. and Krutskikh, E.P. and Novikova, A.G. and Popov, V.N.; Nrf2/are activators improve memory in aged mice via maintaining of mitochondrial quality control of brain and the modulation of gut microbiome; Pharmaceuticals	2021	Excluded	Wrong population
Puri, S. and Shaheen, M. and Grover, B.; Nutrition and cognitive health: A life course approach; Frontiers in Public Health	2023	Excluded	Wrong publication type
Romanenko, M. and Kholin, V. and Koliada, A. and Vaiserman, A.; Nutrition, Gut Microbiota, and Alzheimer's Disease; Frontiers in Psychiatry	2021	Excluded	Wrong publication type
Estrada, J.A. and Contreras, I.; Nutritional modulation of immune and central nervous system homeostasis: The role of diet in development of neuroinflammation and neurological disease; Nutrients	2019	Excluded	Wrong publication type
Song, Z. and Zhang, X. and Hong, M. and Wu, Z. and Luo, S. and Cheng, K.; Oolong tea polyphenols affect the inflammatory response to improve cognitive function by regulating gut microbiota; Journal of Functional Foods	2023	Excluded	Wrong population
Krishna, G.; Oral supplements of inulin during gestation offsets rotenone-induced oxidative impairments and neurotoxicity in maternal and prenatal rat brain; Biomedicine and Pharmacotherapy	2018	Excluded	Wrong publication type
Carregosa, D. and Mota, S. and Ferreira, S. and Alves-Dias, B. and Loncarevic-Vasiljkovic, N. and Crespo, C.L. and Menezes, R. and Teodoro, R. and Santos, C.N.; Overview of beneficial effects of (Poly)phenol metabolites in the context of neurodegenerative diseases on model organisms; Nutrients	2021	Excluded	Wrong publication type

Shao, A. and Lin, D. and Wang, L. and Tu, S. and Lenahan, C. and Zhang, J.; Oxidative stress at the crossroads of aging, stroke and depression; Aging and Disease	2020	Excluded	Wrong publication type
Wang, J. and Yang, Y. and Shi, Y. and Wei, L. and Gao, L. and Liu, M.; Oxidized/unmodified-polyethylene microplastics neurotoxicity in mice: Perspective from microbiota-gut-brain axis; Environment International	2024	Excluded	Wrong population
Scassellati, C. and Galoforo, A.C. and Bonvicini, C. and Esposito, C. and Ricevuti, G.; Ozone: a natural bioactive molecule with antioxidant property as potential new strategy in aging and in neurodegenerative disorders; Ageing Research Reviews	2020	Excluded	Wrong population
Cao B and Zeng MN and Hao FX and Hao ZY and Zhang ZK and Liang XW and Wu YY and Zhang YH and Feng WS and Zheng XK; P-coumaric acid ameliorates Aβ(25-35)-induced brain damage in mice by modulating gut microbiota and serum metabolites.; Biomedicine & pharmacotherapy = Biomedecine & pharmacotherapie	2023	Excluded	Wrong population
Zhang Y and Qian W and Zhang Y and Ma Y and Qian J and Li J and Wei X and Long Y and Wan X; Pediococcus acidilactici reduces tau pathology and ameliorates behavioral deficits in models of neurodegenerative disorders.; Cell communication and signaling : CCS	2024	Excluded	Wrong population
Strac, D.S. and Konjevod, M. and Sagud, M. and Perkovic, M.N. and Erjavec, G.N. and Vuic, B. and Simic, G. and Vukic, V. and Mimica, N. and Pivac, N.; Personalizing the care and treatment of alzheimer's disease: An overview; Pharmacogenomics and Personalized Medicine	2021	Excluded	Wrong publication type
Du, XX and Amin, N and Xu, LH and Botchway, BOA and Zhang, B and Fang, MR; Pharmacological intervention of curcumin via the NLRP3 inflammasome	2023	Excluded	Wrong publication type

in ischemic stroke; FRONTIERS IN PHARMACOLOGY			
Zhang, N. and Guo, P. and Zhao, Y. and Qiu, X. and Shao, S. and Liu, Z. and Gao, Z.; Pharmacological mechanisms of puerarin in the treatment of Parkinson's disease: An overview; Biomedicine and Pharmacotherapy	2024	Excluded	Wrong publication type
Mandalari G and Barreca D and Gervasi T and Roussell MA and Klein B and Feeney MJ and Carughi A; Pistachio Nuts (Pistacia vera L.): Production, Nutrients, Bioactives and Novel Health Effects.; Plants (Basel, Switzerland)	2021	Excluded	Wrong population
Martin, M. and Boulaire, M. and Lucas, C. and Peltier, A. and Pourtau, L. and Gaudout, D. and Layé, S. and Pallet, V. and Joffre, C. and Dinel, A.-L.; Plant Extracts and ω-3 Improve Short-Term Memory and Modulate the Microbiota–Gut–Brain Axis in D-galactose Model Mice; Journal of Nutrition	2024	Excluded	Wrong population
Shanmugam, H and Ganguly, S and Priya, B; Plant food bioactives and its effects on gut microbiota profile modulation for better brain health and functioning in Autism Spectrum Disorder individuals: A review; FOOD FRONTIERS	2022	Excluded	Wrong publication type
Zeng, M.-N. and Cao, B. and Feng, A.-Z. and Guo, P.-L. and Liu, M. and Zhang, Y.-H. and Li, M. and Zheng, X.-K.; Platycladi Semen oil ameliorates Aβ25-35-induced brain injury in mice based on network pharmacology and gut microbiota; Zhongguo Zhongyao Zazhi	2023	Excluded	Wrong population
Potì, F. and Santi, D. and Spaggiari, G. and Zimetti, F. and Zanotti, I.; Polyphenol health effects on cardiovascular and neurodegenerative disorders: A review and meta-analysis; International Journal of Molecular Sciences	2019	Excluded	Wrong publication type
Johnson, S.L. and Kirk, R.D. and Dasilva, N.A. and Ma, H. and Seeram, N.P. and Bertin, M.J.; Polyphenol microbial metabolites exhibit gut and	2019	Excluded	Wrong population

blood–brain barrier permeability and protect murine microglia against lps-induced inflammation; Metabolites			
Morris, G and Gamage, E and Travica, N and Berk, M and Jacka, FN and O'Neil, A and Puri, BK and Carvalho, AF and Bortolasci, CC and Walder, K and Marx, W; Polyphenols as adjunctive treatments in psychiatric and neurodegenerative disorders: Efficacy, mechanisms of action, and factors influencing inter-individual response; FREE RADICAL BIOLOGY AND MEDICINE	2021	Excluded	Wrong publication type
Reddy VP and Aryal P and Robinson S and Rafiu R and Obrenovich M and Perry G; Polyphenols in Alzheimer's Disease and in the Gut-Brain Axis.; Microorganisms	2020	Excluded	Wrong publication type
Filosa, S. and Di Meo, F. and Crispi, S.; Polyphenols-gut microbiota interplay and brain neuromodulation; Neural Regeneration Research	2018	Excluded	Wrong publication type
Liu, Y. and Li, H. and Ren, P. and Che, Y. and Zhou, J. and Wang, W. and Yang, Y. and Guan, L.; Polysaccharide from Flammulina velutipes residues protects mice from Pb poisoning by activating Akt/GSK3β/Nrf-2/HO-1 signaling pathway and modulating gut microbiota; International Journal of Biological Macromolecules	2023	Excluded	Wrong population
Ai, X. and Yu, P. and Li, X. and Lai, X. and Yang, M. and Liu, F. and Luan, F. and Meng, X.; Polysaccharides from Spirulina platensis: Extraction methods, structural features and bioactivities diversity; International Journal of Biological Macromolecules	2023	Excluded	Wrong publication type
Park, S.K. and Kang, J.Y. and Kim, J.M. and Kim, M.J. and Lee, H.L. and Moon, J.H. and Jeong, H.R. and Kim, H.-J. and Chung, M.-Y. and Heo, H.J.; Porphyra tenera Protects against PM2.5-Induced Cognitive Dysfunction with the Regulation of Gut Function; Marine Drugs	2022	Excluded	Wrong population

Silva de Carvalho, T. and Singh, V. and Mohamud Yusuf, A. and Wang, J. and Schultz Moreira, A.R. and Sanchez-Mendoza, E.H. and Sardari, M. and Nascentes Melo, L.M. and Doeppner, T.R. and Kehrmann, J. and Scholtysik, R. and Hitpass, L. and Gunzer, M. and Hermann, D.M.; Post-ischemic protein restriction induces sustained neuroprotection, neurological recovery, brain remodeling, and gut microbiota rebalancing; Brain, Behavior, and Immunity	2022	Excluded	Wrong population
Ross FC and Mayer DE and Horn J and Cryan JF and Del Rio D and Randolph E and Gill CIR and Gupta A and Ross RP and Stanton C and Mayer EA; Potential of dietary polyphenols for protection from age-related decline and neurodegeneration: a role for gut microbiota?; Nutritional neuroscience	2024	Excluded	Wrong publication type
Chen, T. and Jia, F. and Yu, Y. and Zhang, W. and Wang, C. and Zhu, S. and Zhang, N. and Liu, X.; Potential Role of Quercetin in Polycystic Ovary Syndrome and Its Complications: A Review; Molecules	2022	Excluded	Wrong publication type
Zhao, Y. and Zhong, X. and Yan, J. and Sun, C. and Zhao, X. and Wang, X.; Potential roles of gut microbes in biotransformation of natural products: An overview; Frontiers in Microbiology	2022	Excluded	Wrong publication type
Yang Y and Zhou B and Zhang S and Si L and Liu X and Li F; Prebiotics for depression: how does the gut microbiota play a role?; Frontiers in nutrition	2023	Excluded	Wrong publication type
Amalraj, A. and Sukumaran, N.P. and Nair, A. and Gopi, S.; Preparation of a Unique Bioavailable Bacoside Formulation (Cognique®) Using Polar-Nonpolar-Sandwich (PNS) Technology and Its Characterization, In Vitro Release Study, and Proposed Mechanism of Action; Regenerative Engineering and Translational Medicine	2021	Excluded	Wrong population

Tsao, S.-P. and Nurrahma, B.A. and Kumar, R. and Wu, C.-H. and Yeh, T.-H. and Chiu, C.-C. and Lee, Y.-P. and Liao, Y.-C. and Huang, C.-H. and Yeh, Y.-T. and Huang, H.-Y.; Probiotic enhancement of antioxidant capacity and alterations of gut microbiota composition in 6-hydroxydopamin-induced parkinson's disease rats; Antioxidants	2021	Excluded	Wrong population
Cheon, M.-J. and Lim, S.-M. and Lee, N.-K. and Paik, H.-D.; Probiotic properties and neuroprotective effects of lactobacillus buchneri ku200793 isolated from korean fermented foods; International Journal of Molecular Sciences	2020	Excluded	Wrong publication type
Nurrahma, B.A. and Tsao, S.-P. and Wu, C.-H. and Yeh, T.-H. and Hsieh, P.-S. and Panunggal, B. and Huang, H.-Y.; Probiotic Supplementation Facilitates Recovery of 6-OHDA-Induced Motor Deficit via Improving Mitochondrial Function and Energy Metabolism; Frontiers in Aging Neuroscience	2021	Excluded	Wrong population
Taghizadeh Ghassab, F. and Shamlou Mahmoudi, F. and Taheri Tinjani, R. and Emami Meibodi, A. and Zali, M.R. and Yadegar, A.; Probiotics and the microbiota-gut-brain axis in neurodegeneration: Beneficial effects and mechanistic insights; Life Sciences	2024	Excluded	Wrong publication type
Tan, A.H. and Hor, J.W. and Chong, C.W. and Lim, S.-Y.; Probiotics for Parkinson's disease: Current evidence and future directions; JGH Open	2021	Excluded	Wrong publication type
Lee, S. and Eom, S. and Lee, J. and Pyeon, M. and Kim, K. and Choi, K.Y. and Lee, J.H. and Shin, D.J. and Lee, K.H. and Oh, S. and Lee, J.H.; Probiotics that Ameliorate Cognitive Impairment through Anti-Inflammation and Anti-Oxidation in Mice; Food Science of Animal Resources	2023	Excluded	Wrong population
Xue, Y. and Zhang, Y.-N. and Wang, M. and Fu, H.-Y. and Mao, Y.-C. and Hu, M. and Sun, M.-T. and Guo, H.-G. and Cao,	2024	Excluded	Wrong population

L. and Feng, C.-Z.; Prolonged oral intake of green tea polyphenols attenuates delirium-like behaviors in mice induced by anesthesia/surgery; Heliyon			
Yang, Y. and Xiao, G. and Cheng, P. and Zeng, J. and Liu, Y.; Protective Application of Chinese Herbal Compounds and Formulae in Intestinal Inflammation in Humans and Animals; Molecules	2023	Excluded	Wrong publication type
Xie, J. and Song, W. and Liang, X. and Zhang, Q. and Shi, Y. and Liu, W. and Shi, X.; Protective effect of quercetin on streptozotocin-induced diabetic peripheral neuropathy rats through modulating gut microbiota and reactive oxygen species level; Biomedicine and Pharmacotherapy	2020	Excluded	Wrong population
Alfawaz, H.A. and El-Ansary, A. and Al-Ayadhi, L. and Bhat, R.S. and Hassan, W.M.; Protective Effects of Bee Pollen on Multiple Propionic Acid-Induced Biochemical Autistic Features in a Rat Model; Metabolites	2022	Excluded	Wrong population
Chen, S.-Y. and Weng, M.-H. and Li, Z.-Y. and Wang, G.-Y. and Yen, G.-C.; Protective effects of camellia and olive oils against cognitive impairment via gut microbiota-brain communication in rats; Food and Function	2022	Excluded	Wrong publication type
Xi, Y. and Li, H. and Yu, M. and Li, X. and Li, Y. and Hui, B. and Zeng, X. and Wang, J. and Li, J.; Protective effects of chlorogenic acid on trimethyltin chloride-induced neurobehavioral dysfunctions in mice relying on the gut microbiota; Food and Function	2022	Excluded	Wrong population
Kilinc, E. and Ankarali, S. and Ayhan, D. and Ankarali, H. and Torun, I.E. and Cetinkaya, A.; Protective effects of long-term probiotic mixture supplementation against pentylenetetrazole-induced seizures, inflammation and oxidative stress in rats; Journal of Nutritional Biochemistry	2021	Excluded	Wrong population

Arslanova, A. and Tarasova, A. and Alexandrova, A. and Novoselova, V. and Shaidullov, I. and Khusnutdinova, D. and Grigoryeva, T. and Yarullina, D. and Yakovleva, O. and Sitdikova, G.; Protective effects of probiotics on cognitive and motor functions, anxiety level, visceral sensitivity, oxidative stress and microbiota in mice with antibiotic-induced dysbiosis; Life	2021	Excluded	Wrong publication type
Dhyani P and Goyal C and Dhull SB and Chauhan AK and Singh Saharan B and Harshita and Duhan JS and Goksen G; Psychobiotics for Mitigation of Neuro-Degenerative Diseases: Recent Advancements.; Molecular nutrition & food research	2024	Excluded	Wrong publication type
Hu, J. and Jiao, W. and Tang, Z. and Wang, C. and Li, Q. and Wei, M. and Song, S. and Du, L. and Jin, Y.; Quercetin inclusion complex gels ameliorate radiation-induced brain injury by regulating gut microbiota; Biomedicine and Pharmacotherapy	2023	Excluded	Wrong outcome
Yang, S. and Zhou, H. and Wang, G. and Zhong, X.-H. and Shen, Q.-L. and Zhang, X.-J. and Li, R.-Y. and Chen, L.-H. and Zhang, Y.-H. and Wan, Z.; Quercetin is protective against short-term dietary advanced glycation end products intake induced cognitive dysfunction in aged ICR mice; Journal of Food Biochemistry	2020	Excluded	Wrong population
Li, B. and Yan, Y. and Zhang, T. and Xu, H. and Wu, X. and Yao, G. and Li, X. and Yan, C. and Wu, L.-L.; Quercetin reshapes gut microbiota homeostasis and modulates brain metabolic profile to regulate depression-like behaviors induced by CUMS in rats; Frontiers in Pharmacology	2024	Excluded	Wrong population
George, N. and Jawaid Akhtar, M. and Al Balushi, K.A. and Alam Khan, S.; Rational drug design strategies for the development of promising multi-target directed indole hybrids as Anti-	2022	Excluded	Wrong publication type

Alzheimer agents; Bioorganic Chemistry			
Wu, W. and Niu, B. and Peng, L. and Chen, Q. and Chen, H. and Chen, H. and Xia, W. and Jin, L. and Simal-Gandara, J. and Gao, H.; Recent advances on the effect of nut consumption on cognitive improvement; Food Frontiers	2023	Excluded	Wrong publication type
Peng, X. and Hao, M. and Zhao, Y. and Cai, Y. and Chen, X. and Chen, H. and Zhang, Y. and Dong, L. and Liu, X. and Ding, C. and Liu, W. and Yang, M. and Luo, Y.; Red ginseng has stronger anti-aging effects compared to ginseng possibly due to its regulation of oxidative stress and the gut microbiota; Phytomedicine	2021	Excluded	Wrong population
Çimen, F. and Polat, H. and Ekici, L.; Regulatory Impact of Polyphenols on Intestinal Microbiota Composition and Neuroprotective Effects of These Compounds; Akademik Gida	2020	Excluded	Wrong publication type
Jiang, X. and Huang, G.; Research progress in the effect of nutritional intervention on cognitive impairment related to Alzheimer's disease; Journal of Shanghai Jiaotong University (Medical Science)	2023	Excluded	Wrong publication type
Bai, X. and Zhao, X. and Liu, K. and Yang, X. and He, Q. and Gao, Y. and Li, W. and Han, W.; Mulberry Leaf Compounds and Gut Microbiota in Alzheimer's Disease and Diabetes: A Study Using Network Pharmacology, Molecular Dynamics Simulation, and Cellular Assays; International Journal of Molecular Sciences	2024	Included	Not a RCT
Zhang, L. and Lang, F. and Feng, J. and Wang, J.; Review of the therapeutic potential of Forsythiae Fructus on the central nervous system: Active ingredients and mechanisms of action; Journal of Ethnopharmacology	2024	Excluded	Wrong publication type
Micheli, L. and Bertini, L. and Bonato, A. and Villanova, N. and Caruso, C. and Caruso, M. and Bernini, R. and Tirone, F.; Role of Hydroxytyrosol and	2023	Excluded	Wrong publication type

Oleuropein in the Prevention of Aging and Related Disorders: Focus on Neurodegeneration, Skeletal Muscle Dysfunction and Gut Microbiota; Nutrients			
Fang, S. and Wu, Z. and Guo, Y. and Zhu, W. and Wan, C. and Yuan, N. and Chen, J. and Hao, W. and Mo, X. and Guo, X. and Fan, L. and Li, X. and Chen, J.; Roles of microglia in adult hippocampal neurogenesis in depression and their therapeutics; Frontiers in Immunology	2023	Excluded	Wrong publication type
Ali AM and Kunugi H; Royal Jelly as an Intelligent Anti-Aging Agent-A Focus on Cognitive Aging and Alzheimer's Disease: A Review.; Antioxidants (Basel, Switzerland)	2020	Excluded	Wrong publication type
Zeng, W. and Wu, A.G. and Zhou, X.-G. and Khan, I. and Zhang, R.L. and Lo, H.H. and Qu, L.Q. and Song, L.L. and Yun, X.Y. and Wang, H.M. and Chen, J. and Ng, J.P.L. and Ren, F. and Yuan, S.Y. and Yu, L. and Tang, Y. and Huang, G.X. and Wong, V.K.W. and Chung, S.K. and Mok, S.W.F. and Qin, D.L. and Sun, H.L. and Liu, L. and Hsiao, W.L.W. and Law, B.Y.K.; Saponins isolated from Radix polygalae extent lifespan by modulating complement C3 and gut microbiota; Pharmacological Research	2021	Excluded	Wrong population
Zhang, S. and Wei, D. and Lv, S. and Wang, L. and An, H. and Shao, W. and Wang, Y. and Huang, Y. and Peng, D. and Zhang, Z.; Scutellarin Modulates the Microbiota-Gut-Brain Axis and Improves Cognitive Impairment in APP/PS1 Mice; Journal of Alzheimer's Disease	2022	Excluded	Wrong publication type
Liu, Q. and Wang, Y. and Wan, Y. and Liang, Y. and Tan, Y. and Wei, M. and Hou, T.; Selenium- and/or Zinc-Enriched Egg Diet Improves Oxidative Damage and Regulates Gut Microbiota in D-Gal-Induced Aging Mice; Nutrients	2024	Excluded	Wrong population

Qiao L and Chen Y and Song X and Dou X and Xu C; Selenium Nanoparticles-Enriched Lactobacillus casei ATCC 393 Prevents Cognitive Dysfunction in Mice Through Modulating Microbiota-Gut-Brain Axis.; International journal of nanomedicine	2022	Excluded	Wrong population
Eid, H.M. and Wright, M.L. and Kumar, N. and Qawasmeh, A. and Hassan, S.T.S. and Mocan, A. and Nabavi, S.M. and Rastrelli, L. and Atanasov, A.G. and Haddad, P.S.; Significance of microbiota in obesity and metabolic diseases and the modulatory potential by medicinal plant and food ingredients; Frontiers in Pharmacology	2017	Excluded	Wrong publication type
Bonfili, L. and Cecarini, V. and Cuccioloni, M. and Angeletti, M. and Berardi, S. and Scarpona, S. and Rossi, G. and Eleuteri, A.M.; SLAB51 Probiotic Formulation Activates SIRT1 Pathway Promoting Antioxidant and Neuroprotective Effects in an AD Mouse Model; Molecular Neurobiology	2018	Excluded	Wrong population
Shi, H. and Deng, X. and Ji, X. and Liu, N. and Cai, H.; Sources, dynamics in vivo, and application of astaxanthin and lutein in laying hens: A review; Animal Nutrition	2023	Excluded	Wrong population
Song, M. and Fan, X.; Systemic Metabolism and Mitochondria in the Mechanism of Alzheimer's Disease: Finding Potential Therapeutic Targets; International Journal of Molecular Sciences	2023	Excluded	Wrong publication type
Chu, Z. and Han, S. and Luo, Y. and Zhou, Y. and Zhu, L. and Luo, F.; Targeting gut-brain axis by dietary flavonoids ameliorate aging-related cognition decline: Evidences and mechanisms; Critical Reviews in Food Science and Nutrition	2024	Excluded	Wrong publication type
Alba C MA and Daya M and Franck C; Tart Cherries and health: Current knowledge and need for a better understanding of the fate of phytochemicals in the human	2019	Excluded	Wrong population

gastrointestinal tract.; Critical reviews in food science and nutrition			
Mao, M. and Cao, X. and Liang, Y. and Li, Q. and Chen, S. and Zhou, L. and Zhang, Y. and Guo, Y.; Neuroprotection of rhubarb extract against cerebral ischaemia-reperfusion injury via the gut-brain axis pathway; Phytomedicine	2024	Included	Not a RCT
Katunina, E.A. and Semenova, A.M. and Katunin, D.A.; The complex effect of polyphenols on the gut microbiota and triggers of neurodegeneration in Parkinson's disease; Zhurnal Nevrologii i Psihiatrii imeni S.S. Korsakova	2024	Excluded	Wrong publication type
Yang, X. and Yu, B. and Song, C. and Feng, C. and Zhang, J. and Wang, X. and Cheng, G. and Yang, R. and Wang, W. and Zhu, Y.; The Effect of Long-Term Moderate Static Magnetic Field Exposure on Adult Female Mice; Biology	2022	Excluded	Wrong population
Barber, T.M. and Kabisch, S. and Pfeiffer, A.F.H. and Weickert, M.O.; The Effects of the Mediterranean Diet on Health and Gut Microbiota; Nutrients	2023	Excluded	Wrong publication type
Buga, A.M. and Padureanu, V. and Riza, A.-L. and Oancea, C.N. and Albu, C.V. and Nica, A.D.; The Gut–Brain Axis as a Therapeutic Target in Multiple Sclerosis; Cells	2023	Excluded	Wrong publication type
Carloni, S. and Rescigno, M.; The gut-brain vascular axis in neuroinflammation; Seminars in Immunology	2023	Excluded	Wrong publication type
Leyrolle, Q. and Prado-Perez, L. and Layé, S.; The gut-derived metabolites as mediators of the effect of healthy nutrition on the brain; Frontiers in Nutrition	2023	Excluded	Wrong publication type
Casani-Cubel, J. and Benlloch, M. and Sanchis-Sanchis, C.E. and Marin, R. and Lajara-Romance, J.M. and Orti, J.E.L.R.; The impact of microbiota on the pathogenesis of amyotrophic lateral sclerosis and the possible benefits of polyphenols. An overview; Metabolites	2021	Excluded	Wrong publication type

Kingsbury, M.A. and Bilbo, S.D.; The inflammatory event of birth: How oxytocin signaling may guide the development of the brain and gastrointestinal system; Frontiers in Neuroendocrinology	2019	Excluded	Wrong outcome
Zhang, Y. and Yu, W. and Zhang, L. and Wang, M. and Chang, W.; The Interaction of Polyphenols and the Gut Microbiota in Neurodegenerative Diseases; Nutrients	2022	Excluded	Wrong publication type
Gong, X. and Li, X. and Bo, A. and Shi, R.-Y. and Li, Q.-Y. and Lei, L.-J. and Zhang, L. and Li, M.-H.; The interactions between gut microbiota and bioactive ingredients of traditional Chinese medicines: A review; Pharmacological Research	2020	Excluded	Wrong publication type
Li, Y. and Mo, T. and Yao, Y.; The mechanism and application prospect of intestinal flora regulating inflammatory response and oxidative stress in depression; Kexue Tongbao/Chinese Science Bulletin	2023	Excluded	Wrong publication type
Xu, Q. and Yao, Y. and Liu, Y. and Zhang, J. and Mao, L.; The mechanism of traditional medicine in alleviating ulcerative colitis: regulating intestinal barrier function; Frontiers in Pharmacology	2023	Excluded	Wrong publication type
Bi, C. and Guo, S. and Hu, S. and Chen, J. and Ye, M. and Liu, Z.; The microbiota–gut–brain axis and its modulation in the therapy of depression: Comparison of efficacy of conventional drugs and traditional Chinese medicine approaches; Pharmacological Research	2022	Excluded	Wrong publication type
Marano, G. and Mazza, M. and Lisci, F.M. and Ciliberto, M. and Traversi, G. and Kotzalidis, G.D. and De Berardis, D. and Laterza, L. and Sani, G. and Gasbarrini, A. and Gaetani, E.; The Microbiota–Gut–Brain Axis: Psychoneuroimmunological Insights; Nutrients	2023	Excluded	Wrong publication type

Zhang, J. and Lyu, A. and Wang, C.; The molecular insights of bile acid homeostasis in host diseases; Life Sciences	2023	Excluded	Wrong publication type
Wang, H. and Zhao, T. and Liu, Z. and Ma, J. and Li, X. and Huang, X. and Li, B.; The neuromodulatory effects of flavonoids and gut Microbiota through the gut-brain axis; Frontiers in Cellular and Infection Microbiology	2023	Excluded	Wrong publication type
Zhang, Z. and Zhang, Y. and Li, J. and Fu, C. and Zhang, X.; The neuroprotective effect of tea polyphenols on the regulation of intestinal flora; Molecules	2021	Excluded	Wrong publication type
Annunziata, G. and Sureda, A. and Orhan, I.E. and Battino, M. and Arnone, A. and Jiménez-García, M. and Capó, X. and Cabot, J. and Sanadgol, N. and Giampieri, F. and Tenore, G.C. and Kashani, H.R.K. and Silva, A.S. and Habtemariam, S. and Nabavi, S.F. and Nabavi, S.M.; The neuroprotective effects of polyphenols, their role in innate immunity and the interplay with the microbiota; Neuroscience and Biobehavioral Reviews	2021	Excluded	Wrong publication type
Griñán-Ferré, C. and Bellver-Sanchis, A. and Izquierdo, V. and Corpas, R. and Roig-Soriano, J. and Chillón, M. and Andres-Lacueva, C. and Somogyvári, M. and Sőti, C. and Sanfeliu, C. and Pallàs, M.; The pleiotropic neuroprotective effects of resveratrol in cognitive decline and Alzheimer's disease pathology: From antioxidant to epigenetic therapy; Ageing Research Reviews	2021	Excluded	Wrong publication type
Farhan M and Faisal M; The Potential Role of Polyphenol Supplementation in Preventing and Managing Depression: A Review of Current Research.; Life (Basel, Switzerland)	2024	Excluded	Wrong publication type
Babaei, F. and Navidi-Moghaddam, A. and Naderi, A. and Ghafghazi, S. and Mirzababaei, M. and Dargahi, L. and Mohammadi, G. and Nassiri-Asl, M.;	2024	Excluded	Wrong population

The preventive effects of Saccharomyces boulardii against oxidative stress induced by lipopolysaccharide in rat brain; Heliyon			
Zeng, L. and Xiang, R. and Fu, C. and Qu, Z. and Liu, C.; The Regulatory effect of chlorogenic acid on gut-brain function and its mechanism: A systematic review; Biomedicine and Pharmacotherapy	2022	Excluded	Wrong publication type
Niu, L. and Hou, Y. and Jiang, M. and Bai, G.; The rich pharmacological activities of Magnolia officinalis and secondary effects based on significant intestinal contributions; Journal of Ethnopharmacology	2021	Excluded	Wrong publication type
Ferrari, S. and Galla, R. and Mulè, S. and Rosso, G. and Brovero, A. and Macchi, V. and Ruga, S. and Uberti, F.; The Role of Bifidobacterium bifidum novaBBF7, Bifidobacterium longum novaBLG2 and Lactobacillus paracasei TJB8 to Improve Mechanisms Linked to Neuronal Cells Protection against Oxidative Condition in a Gut-Brain Axis Model; International Journal of Molecular Sciences	2023	Excluded	Wrong population
Angeloni, C. and Businaro, R. and Vauzour, D.; The role of diet in preventing and reducing cognitive decline; Current Opinion in Psychiatry	2020	Excluded	Wrong publication type
Xiong, R.-G. and Li, J. and Cheng, J. and Zhou, D.-D. and Wu, S.-X. and Huang, S.-Y. and Saimaiti, A. and Yang, Z.-J. and Gan, R.-Y. and Li, H.-B.; The Role of Gut Microbiota in Anxiety, Depression, and Other Mental Disorders as Well as the Protective Effects of Dietary Components; Nutrients	2023	Excluded	Wrong publication type
Chen, F. and Zhang, L. and Liu, Y. and Zhang, A. and Wang, W.; Resveratrol alleviates perinatal methylmercury-induced neurobehavioral impairments by modulating the gut microbiota composition and neurotransmitter disturbances; Environmental Toxicology	2024	Included	Animals

Monti K and Conkright MW and Eagle SR and Lawrence DW and Dretsch LM; The role of nutrition in mild traumatic brain injury rehabilitation for service members and veterans.; NeuroRehabilitation	2024	Excluded	Wrong publication type
Yoo S and Jung SC and Kwak K and Kim JS; The Role of Prebiotics in Modulating Gut Microbiota: Implications for Human Health.; International journal of molecular sciences	2024	Excluded	Wrong publication type
Pasinetti, G.M. and Singh, R. and Westfall, S. and Herman, F. and Faith, J. and Ho, L.; The Role of the Gut Microbiota in the Metabolism of Polyphenols as Characterized by Gnotobiotic Mice; Advances in Alzheimer's Disease	2022	Excluded	Wrong population
Zhou, J.-C. and Li, H.-L. and Zhou, Y. and Li, X.-T. and Yang, Z.-Y. and Tohda, C. and Komatsu, K. and Piao, X.-H. and Ge, Y.-W.; The roles of natural triterpenoid saponins against Alzheimer's disease; Phytotherapy Research	2023	Excluded	Wrong publication type
Xia, W. and Liu, B. and Tang, S. and Yasir, M. and Khan, I.; The science behind TCM and Gut microbiota interaction—their combinatorial approach holds promising therapeutic applications; Frontiers in Cellular and Infection Microbiology	2022	Excluded	Wrong publication type
Fanton, S. and Cardozo, L.F.M.F. and Combet, E. and Shiels, P.G. and Stenvinkel, P. and Vieira, I.O. and Narciso, H.R. and Schmitz, J. and Mafra, D.; The sweet side of dark chocolate for chronic kidney disease patients; Clinical Nutrition	2021	Excluded	Wrong publication type
Sathyasaikumar, K.V. and Blanco-Ayala, T. and Zheng, Y. and Schwieler, L. and Erhardt, S. and Tufvesson-Alm, M. and Poeggeler, B. and Schwarcz, R.; The Tryptophan Metabolite Indole-3-Propionic Acid Raises Kynurenic Acid Levels in the Rat Brain In Vivo;	2024	Excluded	Wrong population

International Journal of Tryptophan Research			
Alvarenga, L. and Cardozo, L.F.M.F. and Borges, N.A. and Chermut, T.R. and Ribeiro, M. and Leite, M. and Shiels, P.G. and Stenvinkel, P. and Mafra, D.; To bee or not to bee? The bee extract propolis as a bioactive compound in the burden of lifestyle diseases; Nutrition	2021	Excluded	Wrong publication type
Shipelin, V.A. and Skiba, E.A. and Budayeva, V.V. and Shumakova, A.A. and Kolobanov, A.I. and Sokolov, I.E. and Maisaya, K.Z. and Guseva, G.V. and Trusov, N.V. and Masyutin, A.G. and Delegan, Y.A. and Kocharovskaya, Y.N. and Bogun, A.G. and Gmoshinski, I.V. and Khotimchenko, S.A. and Nikityuk, D.B.; Toxicological Characteristics of Bacterial Nanocellulose in an In Vivo Experiment—Part 1: The Systemic Effects; Nanomaterials	2024	Excluded	Wrong publication type
Li, W. and Zhang, L. and He, P. and Li, H. and Pan, X. and Zhang, W. and Xiao, M. and He, F.; Traditional uses, botany, phytochemistry, and pharmacology of Lonicerae japonicae flos and Lonicerae flos: A systematic comparative review; Journal of Ethnopharmacology	2024	Excluded	Wrong publication type
Neufeld, P.M. and Nettersheim, R.A. and Matschke, V. and Vorgerd, M. and Stahlke, S. and Theiss, C.; Unraveling the gut-brain axis: the impact of steroid hormones and nutrition on Parkinson's disease; Neural Regeneration Research	2024	Excluded	Wrong publication type
Guo, R. and Pang, J. and Zhao, J. and Xiao, X. and Li, J. and Li, J. and Wang, W. and Zhou, S. and Zhao, Y. and Zhang, Z. and Chen, H. and Yuan, T. and Wu, S. and Liu, Z.; Unveiling the neuroprotective potential of dietary polysaccharides: a systematic review; Frontiers in Nutrition	2023	Excluded	Wrong publication type
Zhang, X. and Wang, J. and Zhang, T. and Li, S. and Liu, J. and Li, M. and Lu, J. and Zhang, M. and Chen, H.; Updated Progress on Polysaccharides with Anti-	2024	Excluded	Wrong publication type

Diabetic Effects through the Regulation of Gut Microbiota: Sources, Mechanisms, and Structure–Activity Relationships; Pharmaceuticals			
Wojciechowska, O. and Kujawska, M.; Urolithin A in Health and Diseases: Prospects for Parkinson's Disease Management; Antioxidants	2023	Excluded	Wrong publication type
Rong, Z.-J. and Cai, H.-H. and Wang, H. and Liu, G.-H. and Zhang, Z.-W. and Chen, M. and Huang, Y.-L.; Ursolic Acid Ameliorates Spinal Cord Injury in Mice by Regulating Gut Microbiota and Metabolic Changes; Frontiers in Cellular Neuroscience	2022	Excluded	Wrong publication type
Thi Ngoc, A.P. and Zahoor, A. and Kim, D.G. and Yang, S.H.; Using Synbiotics as a Therapy to Protect Mental Health in Alzheimer's Disease; Journal of Microbiology and Biotechnology	2024	Excluded	Wrong publication type
Li, S. and Liang, T. and Zhang, Y. and Huang, K. and Yang, S. and Lv, H. and Chen, Y. and Zhang, C. and Guan, X.; Vitexin alleviates high-fat diet induced brain oxidative stress and inflammation via anti-oxidant, anti-inflammatory and gut microbiota modulating properties; Free Radical Biology and Medicine	2021	Excluded	Wrong population
Martini D and Negrini L and Marino M and Riso P and Del Bo C and Porrini M; What Is the Current Direction of the Research on Carotenoids and Human Health? An Overview of Registered Clinical Trials.; Nutrients	2022	Excluded	Wrong publication type
Simpson T and Deleuil S and Echeverria N and Komanduri M and Macpherson H and Suo C and Gondalia S and Fard MT and Pipingas A and Scholey A and Stough C; The Australian Research Council Longevity Intervention (ARCLI) study protocol (ANZCTR12611000487910) addendum: neuroimaging and gut microbiota protocol.; Nutrition journal	2019	Included	Not a RCT
Choneva, M. and Shishmanova-Doseva, M. and Dimov, I. and Boyanov, K. and Dimitrov, I. and Vlaykova, T. and	2022	Excluded	Wrong population

Georgieva, K. and Hrischev, P. and Bivolarska, A.; Xylooligosaccharides and aerobic training regulate metabolism and behavior in rats with streptozotocin-induced type 1 diabetes; Open Medicine (Poland)			
Skalny, A.V. and Aschner, M. and Santamaria, A. and Filippini, T. and Gritsenko, V.A. and Tizabi, Y. and Zhang, F. and Guo, X. and Rocha, J.B.T. and Tinkov, A.A.; The Role of Gut Microbiota in the Neuroprotective Effects of Selenium in Alzheimer's Disease; Molecular Neurobiology	2024	Included	Not a RCT
Wood E and Hein S and Mesnage R and Fernandes F and Abhayaratne N and Xu Y and Zhang Z and Bell L and Williams C and Rodriguez-Mateos A; Wild blueberry (poly)phenols can improve vascular function and cognitive performance in healthy older individuals: a double-blind randomized controlled trial.; The American journal of clinical nutrition	2023	Included	RCT
Merino del Portillo, M. and Clemente-Suárez, V.J. and Ruisoto, P. and Jimenez, M. and Ramos-Campo, D.J. and Beltran-Velasco, A.I. and Martínez-Guardado, I. and Rubio-Zarapuz, A. and Navarro-Jiménez, E. and Tornero-Aguilera, J.F.; Nutritional Modulation of the Gut–Brain Axis: A Comprehensive Review of Dietary Interventions in Depression and Anxiety Management; Metabolites	2024	Excluded	Wrong publication type
Ciancarelli, I. and Morone, G. and Iosa, M. and Cerasa, A. and Calabrò, R.S. and Tozzi Ciancarelli, M.G.; Neuronutrition and Its Impact on Post-Stroke Neurorehabilitation: Modulating Plasticity Through Diet; Nutrients	2024	Excluded	Wrong publication type
Reyes, Z. and Stovall, M.C. and Punyamurthula, S. and Longo, M. and Maraganore, D. and Solch-Ottaiano, R.J.; The impact of gut microbiome and diet on post-acute sequelae of SARS-CoV-2 infection; Journal of the Neurological Sciences	2024	Excluded	Wrong publication type

Venable, K.E. and Lee, C.C. and Francis, J.; Addressing Mental Health in Rural Settings: A Narrative Review of Blueberry Supplementation as a Natural Intervention; Nutrients	2024	Excluded	Wrong publication type
Liu, S. and Dai, H. and Wang, R. and Zhang, X.; Dietary flavonoids: Role in preventing neurodegenerative diseases caused by brain aging by modulating the gut microbiota; Food Bioscience	2024	Excluded	Wrong population
Wang, J. and Shen, Y. and Li, L. and Li, L. and Zhang, J. and Li, M. and Qiu, F.; Lycopene attenuates D-galactose-induced memory and behavioral deficits by mediating microbiota-SCFAs-gut-brain axis balance in female CD-1 mice; Journal of Nutritional Biochemistry	2025	Excluded	Wrong population
Kumari, N. and Anand, S. and Shah, K. and Chauhan, N.S.; Recent Insights on the Prospective role of Phytocompounds in Modulating Gut-Brain Axis; Current Pharmacology Reports	2024	Excluded	Wrong publication type
Lu, Y. and Yu, X. and Wang, Z. and Kong, L. and Jiang, Z. and Shang, R. and Zhong, X. and Lv, S. and Zhang, G. and Gao, H. and Yang, N.; Microbiota–gut–brain axis: Natural antidepressants molecular mechanism; Phytomedicine	2024	Excluded	Wrong publication type
Ashique, S. and Mukherjee, T. and Mohanty, S. and Garg, A. and Mishra, N. and Kaushik, M. and Bhowmick, M. and Chattaraj, B. and Mohanto, S. and Srivastava, S. and Taghizadeh-Hesary, F.; Blueberries in focus: Exploring the phytochemical potentials and therapeutic applications; Journal of Agriculture and Food Research	2024	Excluded	Wrong publication type
Yao, L. and Yang, Y. and Yang, X. and Rezaei, M.J.; The Interaction Between Nutraceuticals and Gut Microbiota: a Novel Therapeutic Approach to Prevent and Treatment Parkinson’s Disease; Molecular Neurobiology	2024	Excluded	Wrong publication type
Zhong, H and Xu, J and Yang, MY and Hussain, M and Liu, XF and Feng, FQ	2023	Excluded	Wrong publication type

and Guan, RF; Protective Effect of Anthocyanins against Neurodegenerative Diseases through the Microbial-Intestinal-Brain Axis: A Critical Review; NUTRIENTS			
Fraga, CG and Croft, KD and Kennedy, DO and Tomás-Barberán, FA; The effects of polyphenols and other bioactives on human health; FOOD & FUNCTION	2019	Excluded	Wrong publication type
Ticinesi, A and Mancabelli, L and Carnevali, L and Nouvenne, A and Meschi, T and Del Rio, D and Ventura, M and Sgoifo, A and Angelino, D; Interaction Between Diet and Microbiota in the Pathophysiology of Alzheimer's Disease: Focus on Polyphenols and Dietary Fibers; HANDBOOK OF MICROBIOME AND GUT-BRAIN-AXIS IN ALZHEIMER'S DISEASE	2022	Excluded	Wrong publication type
Magzal, F and Turroni, S and Fabbrini, M and Barone, M and Schorr, AV and Ofran, A and Tamir, S; A personalized diet intervention improves depression symptoms and changes microbiota and metabolite profiles among community-dwelling older adults; FRONTIERS IN NUTRITION	2023	Excluded	Wrong publication type
Zilli, AMH and Zilli, EM; Review of Evidence and Perspectives of Flavonoids on Metabolic Syndrome and Neurodegenerative Disease; PROTEIN AND PEPTIDE LETTERS	2021	Excluded	Wrong publication type
Yan, BW and Chen, ZS and Hu, YY and Yong, Q; Insight in the Recent Application of Polyphenols From Biomass; FRONTIERS IN BIOENGINEERING AND BIOTECHNOLOGY	2021	Excluded	Wrong publication type
Hou, CY and Tain, YL and Yu, HR and Huang, LT; The Effects of Resveratrol in the Treatment of Metabolic Syndrome; INTERNATIONAL JOURNAL OF MOLECULAR SCIENCES	2019	Excluded	Wrong publication type

Micek, A and Jurek, J and Owczarek, M and Guerrera, I and Torrisi, SA and Castellano, S and Grosso, G and Alshatwi, AA and Godos, J; Polyphenol-Rich Beverages and Mental Health Outcomes; ANTIOXIDANTS	2023	Excluded	Wrong publication type
Jayatunga, DPW and Hone, E and Khaira, H and Lunelli, T and Singh, H and Guillemin, GJ and Fernando, B and Garg, ML and Verdile, G and Martins, RN; Therapeutic Potential of Mitophagy-Inducing Microflora Metabolite, Urolithin A for Alzheimer's Disease; NUTRIENTS	2021	Excluded	Wrong publication type
Selhub, EM and Logan, AC and Bested, AC; Fermented foods, microbiota, and mental health: ancient practice meets nutritional psychiatry; JOURNAL OF PHYSIOLOGICAL ANTHROPOLOGY	2014	Excluded	Wrong publication type
Hsu, Y.C.; Huang, Y.Y.; Tsai, S.Y.; Kuo, Y.W.; Lin, J.H.; Ho, H.H.; Chen, J.F.; Hsia, K.C.; Sun, Y. Efficacy of Probiotic Supplements on Brain-Derived Neurotrophic Factor, Inflammatory Biomarkers, Oxidative Stress and Cognitive Function in Patients with Alzheimer's Dementia: A 12-Week Randomized, Double-Blind Active-Controlled Study. Nutrients	2023	Excluded	Wrong population
